# A Herpes Simplex Virus-Derived Replicative Vector Expressing LIF Limits Experimental Demyelinating Disease and Modulates Autoimmunity

**DOI:** 10.1371/journal.pone.0064200

**Published:** 2013-05-20

**Authors:** Michaela Nygårdas, Henrik Paavilainen, Nadine Müther, Claus-Henning Nagel, Matias Röyttä, Beate Sodeik, Veijo Hukkanen

**Affiliations:** 1 Department of Virology, University of Turku, Turku, Finland; 2 Department of Pathology, University of Turku, Turku, Finland; 3 Institute of Virology, Hannover Medical School, Hannover, Germany; Washington University, United States of America

## Abstract

Herpes simplex virus type 1 (HSV-1) has properties that can be exploited for the development of gene therapy vectors. The neurotropism of HSV enables delivery of therapeutic genes to the nervous system. Using a bacterial artificial chromosome (BAC), we constructed an HSV-1(17^+^)-based replicative vector deleted of the neurovirulence gene γ_1_34.5, and expressing leukemia inhibitory factor (LIF) as a transgene for treatment of experimental autoimmune encephalomyelitis (EAE). EAE is an inducible T-cell mediated autoimmune disease of the central nervous system (CNS) and is used as an animal model for multiple sclerosis. Demyelination and inflammation are hallmarks of both diseases. LIF is a cytokine that has the potential to limit demyelination and oligodendrocyte loss in CNS autoimmune diseases and to affect the T-cell mediated autoimmune response. In this study SJL/J mice, induced for EAE, were treated with a HSV-LIF vector intracranially and the subsequent changes in disease parameters and immune responses during the acute disease were investigated. Replicating HSV-LIF and its DNA were detected in the CNS during the acute infection, and the vector spread to the spinal cord but was non-virulent. The HSV-LIF significantly ameliorated the EAE and contributed to a higher number of oligodendrocytes in the brains when compared to untreated mice. The HSV-LIF therapy also induced favorable changes in the expression of immunoregulatory cytokines and T-cell population markers in the CNS during the acute disease. These data suggest that BAC-derived HSV vectors are suitable for gene therapy of CNS disease and can be used to test the therapeutic potential of immunomodulatory factors for treatment of EAE.

## Introduction

Delivering transgenes by viral vectors into the central nervous system (CNS) enables gene therapy of chronic diseases that are characterized by pathological changes in the brain and the spinal cord. Herpes simplex virus (HSV) vectors are good candidates for gene delivery to the nervous system for several reasons: HSV vectors are neurotropic, have a large transgene capacity, infect a variety of cell types and persist lifelong in a latent form in neurons. To construct viral vectors, HSV has been attenuated by deleting essential genes, e.g. those of infected cell protein (ICP) 0, ICP4 and ICP27, or accessory genes such as the neurovirulence gene γ_1_34.5 [1], [2]. HSV mutants deleted of the γ_1_34.5 gene are non-neurovirulent [3]–[5], but nevertheless they have the potential to spread to ependymal cells, oligodendrocytes, astrocytes and also to some neurons in the CNS [6]. The ICP34.5 factor antagonizes in the nervous system the shutoff of protein translation by redirecting the phosphorylated translation factor eIF2α to the host protein phosphatase 1α dephosphorylation [7]. Furthermore, ICP34.5 inhibits the induction of cellular antiviral genes by targeting the TANK-binding kinase (TBK) 1, a major factor of the toll-like receptor-dependent and independent nucleic acid sensing pathways [8]. The ability to replicate in rapidly dividing cells but not in post-mitotic neurons suggests that HSV vectors lacking γ_1_34.5 are promising candidates for CNS tumor therapy [9]–[11]. We and others have previously used Th2 cytokine-expressing, non-replicating or γ_1_34.5-deletion HSV vectors for gene therapy of the autoimmune CNS disease model experimental autoimmune encephalomyelitis (EAE) [12]–[14], the premier animal model for multiple sclerosis (MS).

MS is a chronic inflammatory disease of the CNS during which autoreactive immune cells attack the CNS, oligodendrocytes are lost and the protective myelin sheaths are damaged [15]. The etiology of MS is still poorly understood and the contributing immunological mechanisms are complex. Much of our knowledge on the pathogenesis of MS is derived from studies in animal models. EAE is an inducible T-cell mediated disease of the CNS in rodents [16], with similar symptoms and pathological changes as reported for the human MS. Especially T helper- (Th-) 1 and Th17 cells are important in the pathogenesis [17], whereas Th2 cells might be involved in recovery [18], [19]. Moreover, regulatory CD4^+^CD25^+^ T cells (Tregs) suppress pathogenic autoreactive T cells in EAE [20], thus maintaining the balance between tolerance and immunity [21]. CD8^+^ T cells may also have a regulatory role in EAE [22].

To date, there is no cure for MS, but immunomodulative therapies, such as interferon-β, glatiramer acetate and natalizumab, may delay progression of the disease [23]–[27]. Since destruction of the myelin sheath is a major hallmark of both MS and EAE, neuroprotective and neuroregenerative therapies are urgently needed. The different functions of leukemia inhibitory factor (LIF) in the nervous system make this neurotrophic cytokine a potential candidate for such therapies. LIF is a member of the interleukin (IL)-6 cytokine family, signals through the LIF receptor β (LIFRβ)/glycoprotein 130 (gp130) receptor complex [28], [29], and is expressed in many cell types and organs [30], [31]. LIF promotes the differentiation and survival of oligodendrocytes [32], [33], which are the myelin producing cells of the CNS, and induces an anti-apoptotic state in oligodendrocytes [34]. LIF is also required for normal inflammatory responses to injury in the peripheral nervous system (PNS) and CNS [35]. The effect LIF has on the immune system remains to be elucidated. A role of LIF in Treg function has been suggested and is supported by findings that Tregs secrete high amount of LIF after being activated [36]. In addition, LIF induces FoxP3, the signature transcription factor for Tregs and thus, supports the maturation of Tregs but opposes IL-6-driven Th17 maturation [37].

A neuroprotective role of LIF has also been described in MS and EAE. Vanderlocht et al. [38] have shown that LIF is produced by T lymphocytes and macrophages in inflammatory MS lesions, and that it protects oligodendrocytes against tumor necrosis factor (TNF)-α induced apoptosis. Studies with LIF-knockout animals and with neutralizing antibodies against LIF have shown that endogenous LIF limits demyelination and oligodendrocyte loss [39], [40] and protects axons in EAE [41]. Also systemic and lentiviral administration of LIF ameliorate the disease [39], [42].

We describe here the construction and therapeutic effects of a new LIF-expressing viral vector based on a bacterial artificial chromosome (BAC [43], [44]) of HSV-1 (strain 17^+^) lacking both copies of the neurovirulence gene γ_1_34.5. The HSV-LIF vector significantly ameliorated the course of EAE in SJL/J mice and contributed to a higher number of oligodendrocytes in brains during recovery in comparison to untreated EAE mice. Furthermore, we investigated the mRNA expression patterns of several autoimmunity-related cytokines during the acute disease. The HSV-LIF vector induced favorable changes in the mRNA levels of Th17 and Treg cytokines. The HSV vector by itself also had a beneficial effect on the cytokine balance in EAE. Thus, treatment of EAE with an HSV vector expressing LIF modulates the immune response leading to a favorable outcome.

## Materials and Methods

### Ethics Statement

All animal experiments were performed with approval from the National Animal Experiment Board in Finland (permit ESLH-2008-05175/Ym-23). The recombinant viruses were used under the notification 19/M/07 of the National Board for Gene Technology, Finland.

### Plasmids

An expression cassette for murine LIF was constructed by amplifying nucleotides 218 to 835 of LIF (GenBank NM_008501) from brain samples of healthy mice with primers mLIF ATG-L and mLIF-R (Table S1), followed by cloning of the KpnI and HindIII digested PCR product into the plasmid pCDNA3.1(-)EF1α (a kind gift from Jay Nelson, Oregon Health & Sciences University, Portland, USA). The resulting plasmid pCDNA3.1-EF1αLIF expresses murine LIF from the elongation factor 1α (EF1α) promoter.

### Construction of Recombinant HSV-1(17^+^) BACs

The vector for LIF expression is based on HSV-1 strain 17^+^ and was constructed by mutagenesis of a bacterial artificial chromosome (BAC) [45], [46]. The mutagenesis procedure is described in detail in the Materials and Methods S1 and depicted in Figure S1 and Figure S2. HSV-1(17^+^)Lox-Luc contains a luciferase expression cassette inserted between the viral open reading frames (ORFs) UL55 and UL56. The 3′ copy of the neurovirulence gene γ_1_34.5 was replaced by a kanamycin resistance cassette flanked by FRT sites that was subsequently removed by Flp-recombinase, thus generating HSV-1(17^+^)Lox-Luc-Δγ_1_34.5. Next, the 5′ copy of γ_1_34.5 was replaced by a zeocin resistance cassette resulting in the vector HSV-1(17^+^)Lox-Luc-Δγ_1_34.5-Zeo, or for short HSV-Zeo. For the LIF-expressing vector HSV-1(17^+^)Lox-Luc-Δγ_1_34.5-LIF, designated HSV-LIF, this zeocin resistance cassette was replaced by a construct for expression of LIF from the EF1α promoter, as described above.

### Reconstitution of Recombinant HSV-1(17^+^) from BACs

BAC-DNA was prepared with NucleoBond BAC 100 kit (Macherey & Nagel, Düren, Germany), as described before [45]. Vero cells (ATCC, Manassas, VA, USA) were transfected with approximately 2 µg of BAC-DNA with MBS mammalian transfection kit (Stratagene, La Jolla, CA, USA) and were cultured until viral plaques developed. High titer virus stocks were grown in roller flasks of Vero cells until all cells showed cytopathic effects due to viral infection. Viral stocks were titered and stored at −70°C.

### Analysis of in vitro Transgene Expression

T98G glioblastoma cells (VTT Technical Research Centre, Turku, Finland) and Vero cells were grown in 24-well plates and infected with 0.5 or 5 multiplicity of infection (MOI) of HSV-LIF. As controls, cells were infected with HSV-Zeo or HSV-1 (17^+^) wild-type or were left uninfected. At 24 h and 48 h post infection, supernatants were collected and cells were lysed with a buffer containing 1.2 mM MOPS, pH 8.0, 3.5 mM EDTA, 0.1% NP-40 and a protease inhibitor cocktail tablet (Roche Diagnostics, Mannheim, Germany). Cells were boiled for 5 min at 95°C, and the protein concentration was measured using the BCA protein assay kit (Pierce, ThermoScientific, Erembodegem, Belgium). 30 µg of total protein was separated on a 12.5% sodium dodecyl sulfate-polyacrylamide (SDS) gel. Samples were transferred to Amersham Hybond-P membranes (GE Healthcare Europe GmbH, Munich, Germany). Blots were blocked overnight in phosphate buffered saline (PBS) containing 10% non-fat dry milk and 0.1% Tween-20. Membranes were incubated with the primary rabbit polyclonal antibody LIF M-179, (Santa Cruz biotechnology, Santa Cruz CA, USA), washed with PBS containing 0.1% Tween-20 and incubated with a horseradish peroxidase (HRP) conjugated swine anti-rabbit IgG (Dako, Glostrup, Denmark) and developed with enhanced chemiluminescence (ECL, Pierce, ThermoScientific). The amount of LIF protein expression from the HSV-LIF vector was analyzed from the collected supernatants (samples described above) using mouse LIF Quantikine ELISA kit (R&D Systems Europe, Oxon, UK).

### Mice and EAE Induction

Specific pathogen-free 4–6 week-old female SJL/J OlaHsd mice were obtained from Harlan UK. The mice were maintained at the animal facility of the Microbiological Institute, University of Turku. Experiments were performed under the permit ESLH-2008-05175/Ym-23 of the National Animal Experiment Board in Finland. EAE was induced by immunization with 50 µg proteolipid protein peptide (PLP)_139–151_ (NeoMPS, Strasbourg, France) in both footpads and 200 ng Pertussis toxin (Alexis Biochemicals, Enzo Life Sciences, Plymouth Meeting, PA, USA) intraperitoneally at day 0 and day 1 post induction, as described before [14]. A total of 84 mice were included in the experiment.

### HSV Vector Administration

The recombinant viruses were used under the notification 19/M/07 of the National Board for Gene Technology, Finland. 1×10^7^ PFU of the viral vectors, HSV-LIF or HSV-Zeo were diluted in 10 µl of PBS and administered in anesthetized mice intracranially in the left parietal cerebral cortex (parenchymal), at the anteroposterior level of bregma −0.1 cm, lateral level of 0.1 cm and dorsoventral level of 0.1 cm on day 6 post EAE induction (day 0 of infection). Control groups consisted of untreated EAE mice and EAE mice treated with UV-irradiated vector (HSV-LIF inactivated with UV-light for 30 min, resulting in a reduction of titer by 10^4^-fold, with a residual dose of 5×10^3^ PFU in 10 µl). The different treatment and control groups consisted of 15–26 mice each.

### In vivo Imaging

The spread of the HSV vectors expressing luciferase in the mice was analyzed using the IVIS Imaging System 50 Series apparatus (Xenogen, Caliper Life Sciences, Affligem, Belgium). Mice were injected intraperitoneally with 3 mg in 150 µl of beetle luciferin (Promega, Madison, WI, USA) and anesthetized under isoflurane flow. 5–10 minutes later the mice were moved to the dark chamber of the IVIS 50 apparatus and images were acquired for 1–5 minutes. Images were taken on day 1 to day 5 post infection.

### Dissection and Processing of Tissues

Four to five mice from each group were sacrificed under CO_2_ anesthesia at days 9, 14 and 21 after EAE induction, representing the onset, the acute and the recovery phase of EAE disease, respectively. Mice were perfused with PBS via the left chamber of the heart after lethal CO_2_ anesthesia. Samples were taken from the brains for virus culture and DNA/RNA preparation. The rest of the brains were fixed in formaldehyde solution, 10% phosphate buffered pH 7, (FF-Chemicals Ab, Haukipudas, Finland) for microscopy. Samples from the trigeminal ganglia (TGs) were taken for viral culture and for DNA and RNA extraction. Spinal cord samples were taken for DNA and RNA extraction and the rest of the spinal cords were fixed in formaldehyde solution, 10% phosphate buffered pH 7, for microscopy. 4 µm paraffin sections of brain and spinal cord samples were cut and transferred onto glass microscope slides.

### Neuropathological Examinations

Formaldehyde-fixed 4 µm paraffin brain sections were stained with hematoxylin and eosin (HE-staining). Immunohistochemical stainings were done for HSV-1 (1∶1000, Biogenex, San Ramon, CA, USA), astrocytes (GFAP 1∶2000, Abcam, Cambridge, UK), myelin basic protein (MBP 1∶200, Abcam) and oligodendrocytes (CNPase 1∶10000, Covance, Princeton, NJ, USA). Antigen unmasking was achieved by heat treatment in 10 mM citric acid buffer. Unspecific binding was blocked in normal serum of the species of the secondary antibody and sections were incubated overnight at 4°C with the primary antibody. MBP was visualized using Vectastain (Vector laboratories, Burlingame, CA, USA), GFAP and HSV-1 with Brightvision (Immunologic, Duiven, Netherlands) and CNPase with mouse on mouse-kit (Biocare Medical, Concord, CA, USA). For all antibodies diaminobenzidine (DAB) staining was used.

A neuropathologist (MR) scored the severity of inflammatory infiltrates in the CNS (HE-staining). The scoring was: 0, no infiltration; 1, perivascular infiltration; 2, perivascular inflammatory cuffs; 3, inflammation of the brain substance. Pictures were taken with a Carl Zeiss Axiovert 200M microscope and Zeiss AxioCam camera, using AxioVision Software (Carl Zeiss, Göttingen, Germany).

Oligodendrocytes (CNPase staining) were quantified from the rim of the white matter from corresponding frames of the brain sections of each animal. Four to seven frames were used for each brain section. Positive cells were calculated from photographs using Image J software [47] and normalized to the brain area.

### Viral Culture

Brain and TG samples were homogenized by shaking the samples in tubes with glass beads (Assistant Ø 1 mm, VWR International Oy, Espoo, Finland) at 4°C for 2×45 s with Mikro-dismembrator II device (Medical Braun Ab, Espoo, Finland). The supernatant from the homogenized TG and brain samples were diluted 1∶1 in RPMI medium containing 0.1% BSA before overlaying Vero cells in 6-well culture dishes with 0.5 ml virus dilution. Cells were incubated for 1–2 hours at room temperature before changing the medium to minimum essential medium Eagle (MEM) containing heat inactivated 7% fetal calf serum (FCS) and 20 µg/ml human IgG (Sanquin, Amsterdam, Netherlands). Cells were then incubated for four days at 37°C and 5% CO_2_, fixed in methanol for 3 minutes and stained with 0.1% crystal violet in 2% ethanol. Samples taken on day 21 post induction were incubated in culture medium for five days at 37°C (CO_2_-incubator) to reactivate any latent virus before homogenization and plaque titration.

### DNA and RNA Extraction

Extraction of DNA and RNA has been published previously [14]. Shortly, DNA was extracted from brain, TG and spinal cord samples with EZNA tissue extraction kit (OMEGA, Bio-tek, Norcross, GA, USA) according to manufacturer’s manual. RNA was extracted with Tri Reagent (Molecular Research Center Inc., Cincinnati, Ohio, USA) according to manufacturer’s instructions.

### Reverse Transcription

RNA was treated with DNAse before cDNA transcription: 8 µl RNA sample was incubated with 1×DNAse buffer, 1 unit DNAse and 0.8 units RiboLock RNase inhibitor, all from Fermentas (Fermentas, part of Thermo Fischer Scientific, Waltham, MA, USA) at 37°C for 30 min, whereafter 1 µl EDTA stop-solution was added and samples were incubated for 10 min at 65°C. RNA was transcribed into cDNA as follows: 56 pmol Oligo(dT)_18_ (Fermentas) and 27 µl diethylpyrocarbonate (DEPC)-treated water were mixed and added to the DNAse-treated RNA. The sample mixture was incubated at 70°C for 5 min and then put on ice. M-MLV RT 1×Buffer, 10 mM dNTP and 80 units RevertAid H Minus M-MLV Reverse transcriptase (Fermentas) were then mixed in a PCR clean room and added to the samples. The RT-reaction was carried out in PerkinElmer PCR machine (Waltham, MA, USA) under the following conditions; at 42°C for 60 min followed by 90°C for 5 min whereafter the samples were cooled to 4°C. The cDNA was stored at −70°C and used for quantitative real-time PCR.

### Quantitation of HSV-1 Transcription and Host Cytokine Expression

Quantitative real-time RT-PCR was performed with Rotor-Gene real-time instrument (Corbett Research, Mortlake, Victoria, Australia) as described previously, but using Maxima SYBR Green from Fermentas [14]. Copy numbers were normalized to both β-actin and glyceraldehyde 3-phosphate dehydrogenase (GAPDH). Primer sequences for β-actin, IL-23p19, IFN-γ [48], IL-5, IL-10, IFNβ, IFNα6, IL-17A [14] and HSV-1 gD [49] have been published earlier. For primers recognizing GAPDH, LIF, p-LIF, FoxP3, IL-6, RoRγT, TGF-β, GATA3, T-bet and STAT3, see Table S1.

### Statistical Analysis

Each mouse was investigated daily and given a clinical disease score. The cumulative index, calculated by summing the daily clinical scores and dividing it by the number of animals in each group, was compared for the statistically significant differences between mouse groups and different days. Statistical difference in inflammation scores was analyzed for the difference between treatment and control groups. Cytokine and signature transcription factor mRNA expression after vector treatment in SJL/J mice with EAE were compared to uninfected EAE mice. We used Statview software (Abacus Concepts, Berkeley, CA, USA) to calculate the Mann-Whitney U test results for these experiments. The differences in oligodendrocyte numbers were calculated with the T-test (Statview software).

## Results

### LIF Expression by the HSV Vector

**Figure 1:**
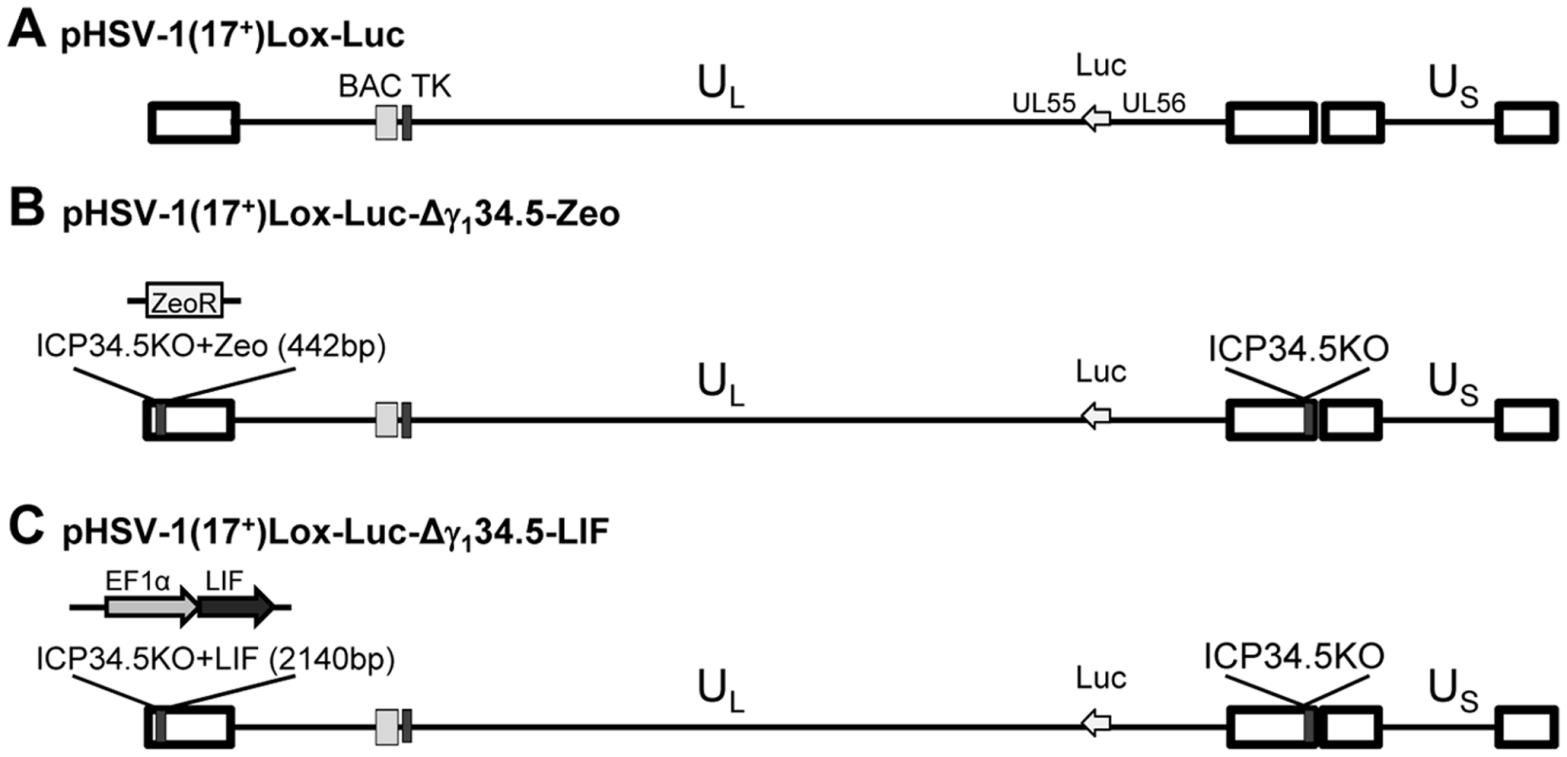
Genome organization and construction of HSV vectors. Schematic representation of the genome organization of HSV-1. (A) In the BAC pHSV-1(17^+^)Lox-Luc, a luciferase cassette under a hCMV promoter was inserted between UL55 and UL56. (B) In pHSV-1(17^+^)Lox-Luc-Δγ_1_34.5-Zeo (designated HSV-Zeo) both copies of γ_1_34.5 have been deleted, with a ZeoR selection cassette remaining in place of the 5′ copy of γ_1_34.5. (C) For pHSV-1(17^+^)Lox-Luc-Δγ_1_34.5-LIF (designated HSV-LIF), the ZeoR cassette was replaced with LIF under control of an EF1alpha promoter.

We constructed the HSV vectors deleted of both copies of the neurovirulence gene γ_1_34.5, and expressing the therapeutic transgene LIF under the control of the elongation factor 1 alpha (EF1α) promoter (HSV-LIF), or expressing the irrelevant control gene Zeo (HSV-Zeo). The transgenes were inserted in the deletion site of the γ_1_34.5 gene in one of the terminal repeat sequences. The vectors also expressed firefly luciferase, enabling *in vivo* imaging during HSV infection. The modifications of the BACs were verified by sequencing. The genomic organization of the HSV-1(17^+^) BAC vectors is presented in Figure 1.

**Figure 2:**
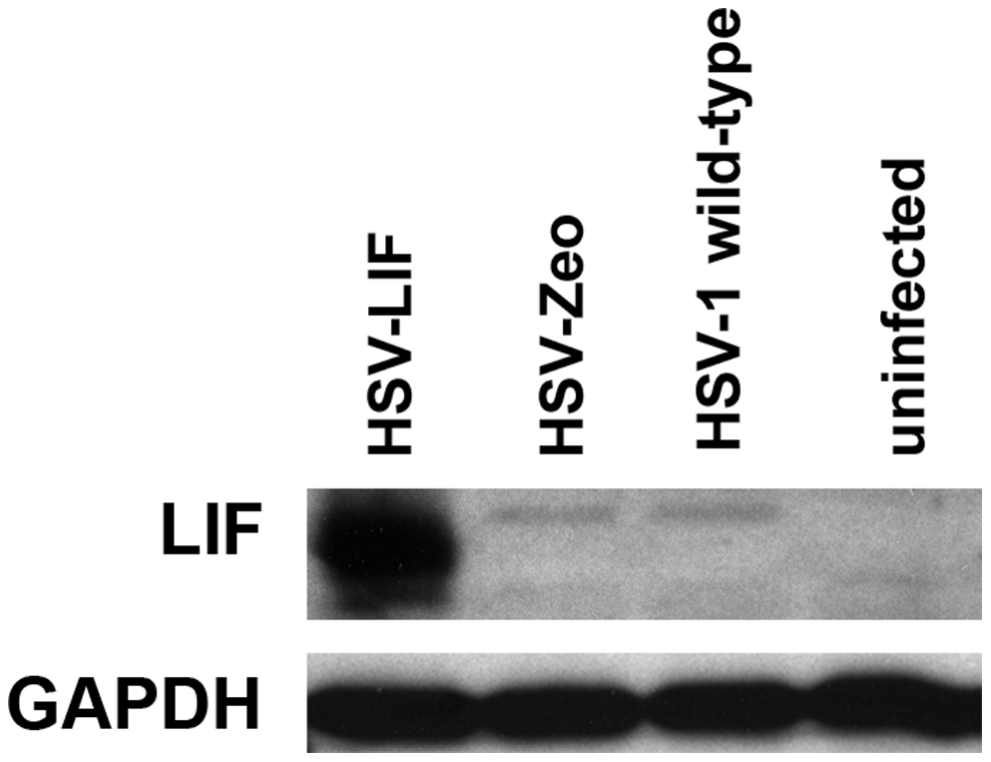
LIF expression from an HSV vector *in vitro*. Immunoblot analysis for transgene LIF expression from the HSV-LIF vector 24 hours after infection of Vero cells at a MOI of 0.5 plaque-forming units per cell. Uninfected cells and cells infected with the control virus (HSV-Zeo) or HSV-1(17^+^) wild-type do not express LIF.

Murine LIF was expressed in cell cultures when infected with the recombinant HSV-LIF as verified by immunoblot (Figure 2) and enzyme-linked immunosorbent assay (ELISA). The amount of recombinant LIF present in infected cell culture samples was 1 pg/µl in the glioblastoma cell line T98G and 2–3 pg/µl in Vero cells at 24 h after infection at a multiplicity of infection (MOI) of 0.5 PFU/cell, meanwhile the backbone virus HSV-Zeo induced no detectable LIF expression. The HSV-LIF grew in cell culture to similar titers as the parental vector, the control vector HSV-Zeo, and HSV-1 wild-type (Figure S3).

### A Replicative HSV-1 Vector Expressing LIF Spreads in the CNS and Ameliorates EAE in SJL/J Mice

We next analyzed the potential therapeutic effects of intracranial administration of the recombinant HSV vectors, expressing either LIF or just a control gene, on the disease course of EAE in SJL/J mice. The study included four groups of SJL/J mice, all induced for EAE: (1) no treatment, (2) UV-irradiated vector (HSV-LIF treated with UV resulting in titer reduction by 10,000-fold), (3) infection with HSV-Zeo, and (4) infection with HSV-LIF. Mice were infected with 1×10^7^ PFU of the respective virus at day 6 post EAE induction. The dose was determined in preliminary studies and the maximum non-toxic deliverable dose chosen (data not shown).

**Figure 3:**
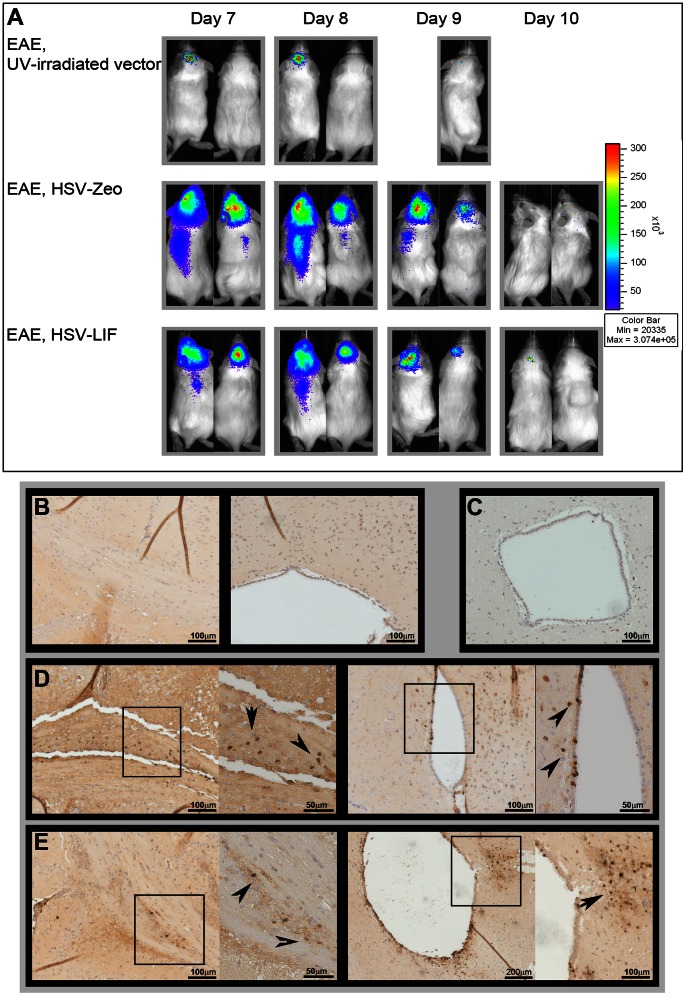
Spread of HSV vectors in the CNS. (A) The HSV vectors expressed luciferase under an hCMV promoter which enables the study of viral spread in the CNS with an IVIS luminometer. 3 mg Luciferin were injected intraperitoneally (i.p.) to anesthetized mice five minutes prior to analysis. The figure shows two representative mice from each treatment group (follow-up of the same mice for all time points). (B-E) Immunohistochemistry for HSV-1 in the brain of untreated EAE mice (B), UV-irradiated vector (C), HSV-Zeo treated mice (D) and HSV-LIF treated mice (E) on day 9 post induction (day 3 post infection). Viral antigens were detected adjacent to the ventricles, in the ependymal cells, and in the corpus callosum in the HSV-vector treated groups (D, E). The arrows in the close-up fields indicate HSV detection. Five mice per group per time point were analyzed for all experimental settings. The scale bars are shown in the figure.

The spread of the virus was studied *in vivo* using the *in vivo* imaging system (IVIS) luminometry. UV-irradiated vector-treated mice only displayed minimal luciferase signal on the day after virus administration (Figure 3A). In mice infected with either the HSV-LIF or the HSV-Zeo, high numbers of photons were detected when imaged at day 1 and 2 post infection (day 7 and 8 post induction). Both vectors had spread to the spinal cord and could even be detected by IVIS in a few mice (Figure 3A) and by PCR in all animals (Table S2). By day 4 post infection (day 10 post induction), the luminescence signal declined and was only detected in a few mice. The distribution of HSV within the brain was studied by immunohistochemistry using an anti-HSV-1 antibody. The vectors could be detected in the brain parenchyma, especially near the third ventricle, the aqueduct and in the corpus callosum, and in ependyma of the ventricles of the brain (Figure 3B–E). We did not observe marked loss of ependymal cells or enlargement of ventricles, which would be attributed to our γ_1_34.5-deletion vectors (Figure S4).

Vector replication was assayed by virus culture for HSV in samples from both the brains and the trigeminal ganglia (TG). The brains of mice infected with HSV-LIF or HSV-Zeo, except for one LIF-treated mouse, contained virus that could be cultured at 3 days post infection (day 9 post induction, data not shown). By day 14 post induction, no replicating vector could be detected anymore. However, HSV DNA was amplified by PCR in brain, trigeminal ganglion and spinal cord samples during the acute infection (Table S2). We also investigated the amount of the transgene LIF DNA (promoter-LIF (p-LIF)) with PCR in the tissue samples. p-LIF was detected in all HSV-LIF treated mice on day 9 post induction, but also in some mice on day 14 and day 21 (Table S2). Thus, HSV DNA and replicating vector were detected during the acute phase of the infection and the vectors had spread also to the spinal cord but were non-virulent.

**Figure 4:**
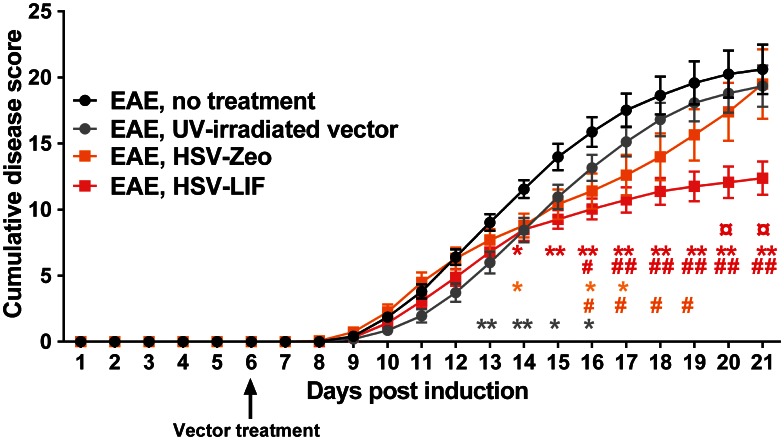
Daily cumulative disease scores. The disease score for each mouse was recorded daily based on the following classification: 0 = healthy; 1 = fur ruffling; 2 = tail atonia; 3 = hind limb paralysis and 4 = tetraparalysis. Mean cumulative disease scores were calculated by summing the daily clinical scores of living mice, divided by the number of animals in the groups. Treatment with HSV-LIF ameliorated the EAE disease course, with significantly lower cumulative disease scores from day 14 on. HSV-Zeo also reduced clinical scores at individual time points. The mice treated with the UV-irradiated vector showed a slightly milder acute disease than untreated mice. Statistically significant differences (Mann-Whitney U test, *p<0.05, **p<0.01 and #p<0.05, ##p<0.01) between HSV-Zeo (orange squares) and HSV-LIF (red squares) treated mice and the untreated (*) or UV-irradiated vector group (#) are indicated with the same colors as the corresponding lines. Significant differences between UV-irradiated vector-treated and uninfected EAE mice are shown as grey asterisks (*). Significant differences between the HSV-LIF and HSV-Zeo groups are indicated with ¤, p<0.05. Results are based on three separate experiments with EAE. The number of mice/group varied depending on the time point, with a total number of mice of (n = 26) for untreated EAE mice, (n = 23) for UV-irradiated vector treatment, (n = 14) for HSV-Zeo treatment and (n = 21) for HSV-LIF treatment. All data are presented as mean ± SEM.

All mice of the 4 different experimental groups developed symptoms typical for EAE within day 9 to day 11 (Table 1). Treatment with the HSV-LIF resulted in lower disease scores than in untreated or UV-irradiated vector-treated EAE mice (Figure S5) and a significant decrease in the cumulative disease score index was seen starting at day 14 post induction (Figure 4). The cumulative index for the HSV-LIF treated group was significantly lower in comparison to all three control groups after disease recovery at day 21 post induction (Figure 4 and Table 1). The control virus HSV-Zeo also lowered the clinical signs significantly at a few time points compared to untreated and UV-irradiated vector-treated EAE mice, but later the disease scores resumed (Figure 4 and Figure S5). Mice were followed for clinical signs for up to 60 days post induction but no relapse occurred (Figure S5).

**Table 1:** Clinical parameters in SJL/J mice induced for EAE, untreated or treated with HSV vectors.

Group	Mean max EAE	Mean day of onset	Cumulative index	EAE
	score (±SEM)^a^	(mean grade >1)^b^	day 21 (±SEM)^c^	mortality
EAE, no treatment	2.79 (0.09)	10.2 (0.24)	20.6 (1.9)	0%
EAE, UV-irradiated vector^d^	2.65 (0.12)	10.4 (0.33)	19.3 (1.6)	0%
EAE, HSV-Zeo	2.68 (0.23)	9.6 (0,25)	19.5 (2.4)	0%
EAE, HSV-LIF	2.39 (0.15)*	10.0 (0.23)	12.4 (1.3) **¤**	0%

a Mean score (±SEM) when mice reached maximal clinical symptoms. The disease score for each mouse was recorded daily based on the following classification: 0 = healthy; 1 = fur ruffling; 2 = tail atonia; 3 = hind limb paralysis and 4 = tetraparalysis.

b Mean day (±SEM) of onset of first clinical symptoms after EAE induction.

c Mean cumulative index (±SEM) was calculated by summing the daily clinical scores of living mice, divided by the number of animals in the groups.

d UV-irradiated vector = UV-inactivated HSV-LIF.

* p = 0.0062 when compared to untreated EAE mice.

¤ p = 0.0072 when compared to untreated EAE mice, p = 0.0077 when compared to UV-irradiated vector-treated mice and p = 0.0414 when compared to HSV-Zeo treated mice.

### Histological Analysis of the CNS

Hematoxylin-eosin and immunohistochemically stained paraffin sections of brain and spinal cord were analyzed for the amount of lymphocyte infiltrates and pathological changes typical for EAE. The samples were scored for the severity of inflammation. In all treatment groups there were inflammatory infiltrates typical for EAE in both the brains and spinal cords, but their amount did not differ significantly between the different groups (Figure S6 and S7).

**Figure 5:**
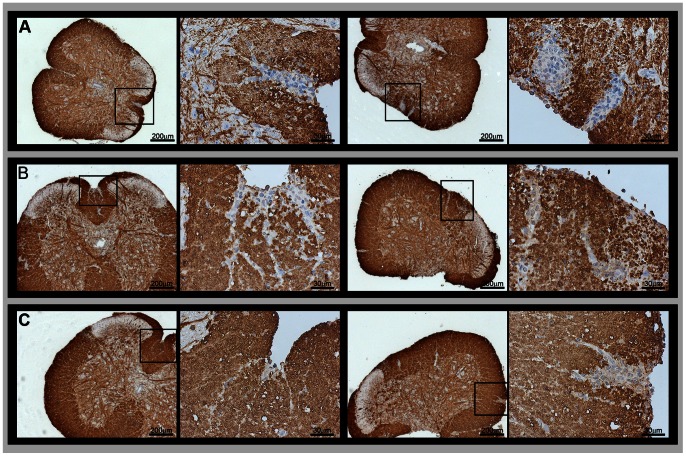
Myelin destruction in the CNS. Detection of myelin by immunohistochemistry for MBP in spinal cords of (A) untreated EAE mice, (B) HSV-Zeo treated EAE mice and (C) HSV-LIF treated EAE mice on day 21 post induction. The figure shows two sections of each group at two magnifications. The squares indicate the areas of inflammatory demyelinating infiltrates at higher magnification. Examples of inflammatory infiltrates disrupting the myelin are shown. Scale bars are shown in the figure.

**Figure 6:**
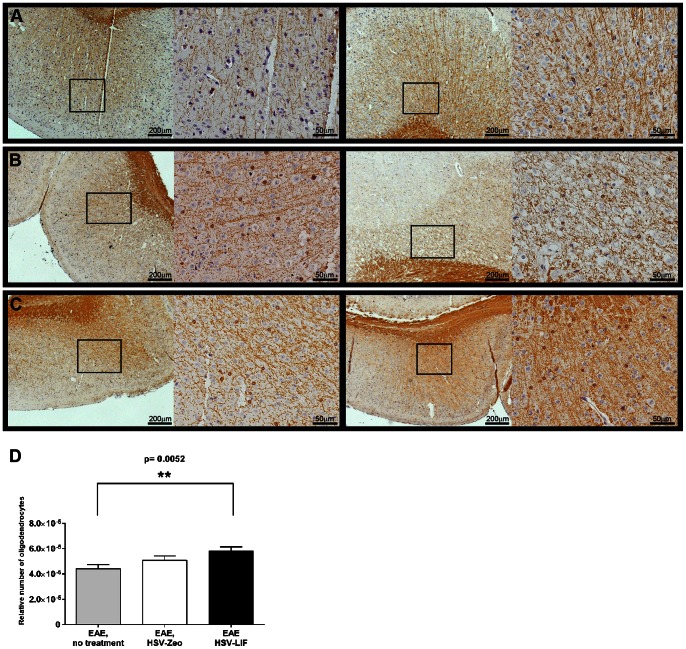
Oligodendrocytes in brains of HSV-treated or untreated EAE mice. (A-C) CNPase antibody labeling showing oligodendrocytes in brains of (A) untreated EAE mice, (B) HSV-Zeo treated EAE mice and (C) HSV-LIF treated EAE mice. (D) Oligodendrocytes were quantified based on the CNPase labeling of brain sections, using four to seven corresponding frames on each section and normalizing to the tissue area. The HSV-LIF treated EAE mice showed a significantly higher amount of oligodendrocytes (p = 0.0052) in brains on day 21 post induction in comparison to untreated mice. Data is presented as mean ± SEM.

Since LIF might induce oligodendrocyte proliferation and thus promote remyelination, we investigated the distribution of the myelin basic protein (MBP) in brain and spinal cord sections of the different groups. The inflammatory demyelinating lesions were found in spinal cords in all EAE groups (Figure 5); while there was some heterogeneity between individual mice within a group, there were no clear differences between the treated and untreated mice. An equal degree of astrocytosis was observed by GFAP staining around infiltrates, the ventricles and in the white matter in all EAE groups (data not shown). The number of oligodendrocytes in corresponding section frames (Figure 6) was significantly higher in brains of HSV-LIF treated mice when compared to non-treated EAE mice on day 21 post induction (p<0.01).

### HSV Vectors Modulate Cytokine Expression in the CNS

**Figure 7:**
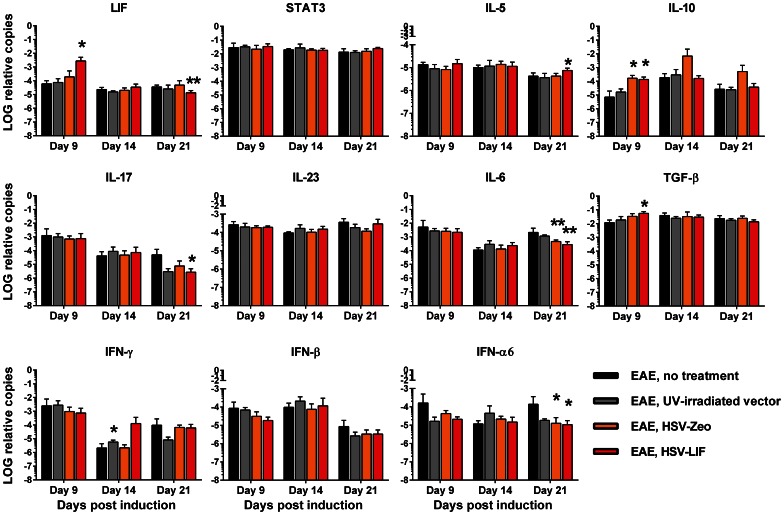
Cytokine mRNA expression in brains of mice with EAE, with or without HSV vector treatment. The expression patterns of LIF, STAT3, IL-5, IL-10, IL-17, IL-23, IL-6, TGF-β, IFN-γ, IFN-β and IFN-α6 mRNA in brains are shown normalized to the housekeeping genes GAPDH and β-actin. Black bar indicates EAE without treatment, grey bar UV-irradiated vector treatment, orange bar treatment with HSV-Zeo and red bar treatment with HSV-LIF. * and ** indicate significant changes (p<0.05 and p<0.01, respectively) in comparison to untreated mice at different time points. Five mice per group were included at each time point. The relative copy number values are shown on a logarithmic scale. SDs are shown.

**Figure 8:**
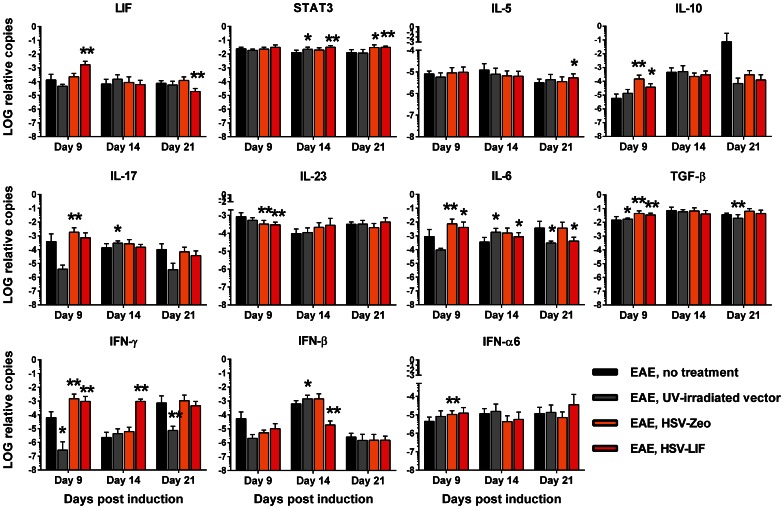
Cytokine mRNA expression in spinal cords of mice with EAE, with or without HSV vector treatment. The expression patterns of LIF, STAT3, IL-5, IL-10, IL-17, IL-23, IL-6, TGF-β, IFN-γ, IFN-β and IFN-α6 mRNA expression in spinal cords are shown normalized to the housekeeping genes GAPDH and β-actin. The black bar indicates EAE without treatment, the grey bar UV-irradiated vector treatment, the orange bar treatment with HSV-Zeo and the red bar treatment with HSV-LIF. * and ** indicate significant changes (p<0.05 and p<0.01 respectively) in comparison to untreated mice at different time points. Five mice per group were included at each time point. The relative copy number values are shown on a logarithmic scale. SDs are shown.

Several autoimmunity and inflammation-related cytokines and T-cell population markers were analyzed from brain and spinal cord samples by quantitative RT-PCR in order to characterize potential HSV vector-induced changes. To represent the onset, acute and recovery phases of EAE disease, we chose three time points: day 9, day 14 and day 21 post induction. Changes in cytokine mRNA levels were detected, especially during the onset and recovery of EAE. The LIF mRNA was strongly expressed after HSV-LIF treatment on day 9 post induction in both brains and spinal cords (Figure 7 and Figure 8). The HSV-LIF did not alter the expression of STAT3, which is involved in LIF receptor signaling, in brain tissue, but a significant increase was detected in spinal cords on day 14 and day 21 post induction (Figure 8). The HSV-Zeo resulted also in increased STAT3 mRNA levels on day 21 (Figure 8). Of the Th2 cytokines, IL-5 was induced in the HSV-LIF treated group on day 21 post induction in the brain and spinal cord (Figure 7 and Figure 8). IL-10, a cytokine also known to be expressed by Tregs, was elevated in both the HSV-Zeo and the HSV-LIF treatment groups during onset of disease (Figure 7 and Figure 8). Furthermore, there was an upregulation of TGF-β in brains of HSV-LIF treated mice, whereas IL-6 tended to decrease (Figure 7), providing a net balance favoring Treg development. For both the HSV-LIF and the control HSV-Zeo, there was a significant decrease in IL-6 mRNA levels in brains during recovery (Figure 7). In spinal cords, TGF-β and IL-6 were upregulated in both the HSV-LIF and the HSV-Zeo groups during onset of disease (Figure 8), but the HSV-LIF later downregulated the IL-6 mRNA expression (Figure 8). We found a significant decrease of IL-17 during recovery in brains of LIF-treated mice (Figure 7). A similar tendency in spinal cords was not significant (Figure 8). The control virus HSV-Zeo upregulated IL-17 mRNA during onset of disease in spinal cords (Figure 8). In spinal cords, the mRNA of the T cell subpopulation signature marker FoxP3 was significantly upregulated in both the HSV-LIF and the HSV-Zeo treated mice on day 9 post induction (Figure S8). This was also the case for T-bet mRNA expression (Figure S8), suggesting a shift from Th17 dominance towards Th1 and Treg populations. In the brains, there were no significant changes in FoxP3, RORγT or T-bet mRNA expression (Figure S8). The cytokine IL-23 was not significantly altered in brains of HSV-treated mice (Figure 7), but a significant decrease could be seen in spinal cords of both HSV-treated groups during onset (Figure 8).

We also studied the interferon responses to the vectors. In the brains, there were no significant changes in the mRNA levels of the two antiviral cytokines IFN-γ or IFN-β (Figure 7). In spinal cords, both HSV-Zeo and HSV-LIF induced a rise in IFN-γ mRNA on day 9 (Figure 8). The HSV-LIF vector also induced IFN-γ on day 14. The UV-irradiated vector did not induce IFN-γ on days 9 and 21 (Figure 8). IFN-β mRNA levels were downregulated in HSV-LIF treated mice on day 14 (Figure 8). The HSV-Zeo and the HSV-LIF also downregulated IFN-α6 in brains during recovery (Figure 7). In spinal cords, the HSV-Zeo treated mice showed an upregulation of IFNα6 on day 9 (Figure 8). The HSV-LIF and UV-irradiated vector-treated mice showed a similar trend. Thus, the therapeutic effect of HSV-LIF was not due to a modulation of the interferon responses, but rather to a shift in the Treg and Th17 cytokine balance.

## Discussion

In this study we constructed replicative vectors derived from HSV-1 (17^+^), deleted of the neurovirulence gene γ_1_34.5, but expressing firefly luciferase for *in vitro* and *in vivo* tracking, using BAC mutagenesis. We investigated the therapeutic effect of an HSV-LIF vector, expressing the neuroprotective cytokine LIF, on acute PLP-induced EAE in SJL/J mice and characterized the therapy-associated changes in the immunological balance in the CNS. Our HSV-LIF expressed LIF and firefly luciferase *in vitro* and *in vivo*. The vectors were detected in the CNS and TG early at days 6–10 post induction and viral DNA persisted in the nervous system until later time points. In addition, the HSV vectors spread from the injection site in the telencephalon to the spinal cord. This capacity of replicative HSV vectors to spread over wider areas is potentially beneficial for therapy of disorders of the whole CNS, while non-replicative viral vectors mostly remain at the injection site [50]. The activity of the HSV vectors in ependymal cells can be advantageous, facilitating spread of the vectors as well as the therapeutic proteins within the CNS via the cerebrospinal fluid [51], but can also be harmful if excess cytotoxicity is associated with the vectors [52]. Ependymal cells were occasionally infected by our HSV vectors (Figure 3), but we did not observe any significant loss of ependymal cells or enlargement of ventricles due to the parenchymal HSV vector administration (Figure S4). HSV-1 (17^+^)-BAC-derived recombinant viruses are suitable for studies in animals, when proper controls are included, as shown previously by others [53]. Our BAC-derived γ_1_34.5-deleted HSV vectors are very attenuated, and cause very little damage to ependymal cells or other components of the CNS upon parenchymal administration. The added attenuation might be due to some small alterations after BAC mutagenesis, such as modification of the *a* sequences and loss of the oriL, as reported for other HSV-1 (17^+^) BACs by Nagel et al [45].

Treatment with HSV-LIF significantly ameliorated acute EAE, as the clinical symptoms were reduced after disease onset, and a significantly lower cumulative disease score was obtained after treatment with HSV-LIF. The HSV-Zeo control also decreased the clinical symptoms to some extent early during the disease. We have previously shown that HSV vectors, deleted of γ_1_34.5, may have a beneficial effect on EAE by themselves [13], [14]. Here, we have focused on the early stages of the EAE, in order to investigate the cytokine profile during the acute phase of the disease, and to compare its progression with our earlier studies using HSV-1 (F) derived Th2-cytokine expressing vectors [13], [14]. Furthermore, in this EAE model the mice did not develop a relapse, even though they were followed for up to 60 days post induction. The lack of relapses in our SJL model could be explained by the younger mice used in our study. Gene therapy with LIF may also have long-term benefits since Slaets et al. [42] have reported that lentiviral LIF-therapy ameliorated EAE during the relapses.

In our PLP-EAE model, we detected inflammatory infiltrates disrupting the myelin in both brain and spinal cords. Acute EAE models do not always result in demyelination during the first phase of clinical disease, and several EAE models with different disease pathogenesis have been developed [54]. In absence of distinct demyelination, it is difficult to determine whether the HSV-LIF affected the de- or remyelination. However, the numbers of oligodendrocytes were higher on day 21 post induction in the HSV-LIF treated mice in comparison to untreated mice. Several studies have indicated a role for LIF in promoting oligodendrocyte survival *in vitro* and after spinal cord injury [32], [33]. Butzkueven et al. have also shown that endogenously administered LIF prevents oligodendrocyte death in EAE [39], [40]. Even though we did not see alterations in myelination, the higher amount of oligodendrocytes detected here could foster remyelination. As astrocytes express LIF, and as LIF itself may regulate GFAP expression and directly activate astrocytes [55], [56], we analyzed the GFAP expression in brain and spinal cord sections. The astrocytes were accumulated around infiltrates and ventricles, as expected in EAE, but there were no significant differences in GFAP expression between the treatment groups (data not shown).

To further investigate the effect of HSV-LIF on the immune response, we studied the mRNA levels of pro- and anti-inflammatory cytokines in the CNS. In previous HSV gene therapy with Th2 cytokine-expressing vectors we have observed changes in T helper cell signature cytokines [14]. Since EAE is considered as a T-cell mediated inflammatory disease, we focused on marker cytokines for different autoimmunity-related T-cell populations. As LIF may affect the Treg/Th17 axis [37], we analyzed the mRNA expression of the markers and cytokines FoxP3, IL-10, TGF-β, IL-6, RoRγT and IL-17. There was indeed a downregulation of IL-17 in the brains during recovery of LIF-treated mice in comparison to untreated mice. Furthermore, TGF-β mRNA expression was induced at early time points whereas IL-6 was downregulated in HSV-LIF treated mice. These cytokine mRNA changes indicate a shift in the Treg/Th17 cell axis of HSV-LIF treated EAE mice. Both HSV-LIF and HSV-Zeo upregulated the IL-10 mRNA expression early in disease and HSV-Zeo also downregulated IL-6 during recovery. These data suggest that also the HSV backbone by itself had a beneficial effect on the cytokine response, even though no significant changes in FoxP3 and RoRγT mRNA expression were seen.

In contrast to the cytokine changes in the brains, the alterations were not as clear-cut in the spinal cords. The HSV-LIF did not significantly alter the IL-17 mRNA expression. IL-6 mRNA expression was upregulated early in disease in HSV treated mice, but later decreased in HSV-LIF treated mice. Both HSV-Zeo and HSV-LIF upregulated TGF-β, IL-10 and FoxP3 mRNA expression during disease onset. Taking together, these data suggest that the HSV vectors induced a shift from Th17 towards Treg responses in our EAE treatment model.

MS and EAE research has long emphasized the Th1/Th2 paradigm, according to which Th2 cytokines are considered to have an alleviating effect on the disease course [19]. We detected only low IL-4 mRNA levels (data not shown), but the HSV-LIF upregulated IL-5, also a Th2 cytokine, during recovery in both brains and spinal cords. The HSV vectors downregulated IL-23 but upregulated IFN-γ mRNA expression in the spinal cords during onset of disease. In line with our previous findings, the HSV vectors did not induce changes in IFN-γ in the brain [14], even though infection with HSV-1 wild-type is known to activate IFN-γ [57]. The upregulated Th1 response in spinal cords, as seen in increased IFN-γ and T-bet mRNA levels, is most likely a beneficial effect of the HSV-1 infection in our EAE model.

The importance of type I IFNs in the recovery from EAE has been reported by several groups [58], [59]. We have also in a previous study detected increased IFN-β and IFN-α mRNA levels in EAE mice treated with HSV vectors expressing Th2 cytokines [14]. The vectors used in the present study did not have this effect. The therapeutic effect of our HSV-LIF vector therefore does not seem to be due to an induction of type 1 interferon, as was the case in earlier HSV-Th2 cytokine therapy. It seems more likely that the shift from Th17 towards Th1 and Treg responses and the higher amount of oligodendrocytes account for the positive effects attenuating the disease course. Our results, collectively, indicate that LIF is a promising candidate as a therapeutic gene and that the replicative HSV-LIF vector induces a trophic and an anti-inflammatory response in EAE, thus supporting the notion that HSV vectors may be of use in gene therapy of CNS diseases.

## Supporting Information

Figure S1 **Repair of UL23 in the HSV-1(17^+^) BAC.** Schematic representation of the mutagenesis procedure for the repair of UL23 gene and the insertion of a Cre/Lox recombination system into pHSV-1(17^+^)blue [45]. After transfection of eukaryotic cells with the resulting BAC pHSV-1(17^+^)Lox, Cre recombinase excises the non-viral sequences (depicted in the green box) from the genome, leaving a single loxP site between the UL22 and UL23 ORFs. The gene structure of the parental virus is shown on the top line and the resulting BAC-derived virus genome is shown at the bottom.Abbreviations: parA-B, repE, oriS: BAC replication origin and regulatory genes. cat: chloramphenicol resistance, tetR: tetracyclin resistance, lacZ: eukaryotic beta-galactosidase expression cassette, loxP: Cre recombinase recognition site, rpsLneo: streptomycin sensitivity/kanamycin resistance counterselection/selection cassette, Cre: eukaryotic Cre recombinase expression cassette. frt: Flp recombinase recognition site.
(TIF)

Figure S2 **Restriction endonuclease patterns of HSV BAC DNA.** Restriction profiles of chosen BAC clones of pHSV-1(17^+^)Lox-Luc, pHSV-1(17^+^)Lox-Luc-Δγ_1_34.5-Zeo, pHSV-1(17^+^)Lox-Luc-Δγ_1_34.5-KanLIF and pHSV-1(17^+^)Lox-Luc-Δγ_1_34.5-LIF. **(A)** Changes in fragment size (kbp) after PstI, XbaI and BamHI restriction digest. L = band shifts in the 5′ area of γ_1_34.5, R = band shifts in the 3′ area of γ_1_34.5, L/R band shifts covering both γ_1_34.5 copies. **(B-C)** Gel elecrophoresis. (LL) = pHSV-1(17^+^)Lox-Luc, (Zeo) = pHSV-1(17^+^)Lox-Luc-Δγ_1_34.5-Zeo, (Kan) = pHSV-1(17^+^)Lox-Luc-Δγ_1_34.5-KanLIF, (LIF) = pHSV-1(17^+^)Lox-Luc-Δγ_1_34.5-LIF. Stars (*) indicate changes in restriction pattern after the deletion of the 3′ γ_1_34.5, (<) indicate changes in restriction pattern after modification of the 5′ γ_1_34.5 of HSV-1. Abbreviations: HSV, herpes simplex virus; Zeo, zeocin resistance cassette; LIF, leukemia inhibitory factor.
(TIF)

Figure S3 **Growth curves of HSV-1(17^+^) wild-type and BAC-derived HSV vectors.** Vero cells (in 6 well-plates) were infected with 5 MOI of respective viruses. Cells were washed at 6 hours post infection to remove the original virus stock. Supernatant samples were taken every six hours. The virus amount in the supernatant was determined with plaque titration.
(TIF)

Figure S4 **Brain ventricular ependymal cells in mice infected with the γ_1_34.5-deleted HSV vectors.** Ependymal cells are shown at the 3^rd^ ventricles in HSV-Zeo and HSV-LIF treated EAE mice. Untreated EAE mice were included as controls. The sections in the left column are parallel to sections positive for HSV in immunohistochemical stainings (Figure 3), representing day 9 post induction (day 3 post infection). The right side panel shows brains representing day 21 post induction (day 15 post infection). No clear difference in ependymal cell line was detected between the treatment groups. The day 21 sections shown for HSV-Zeo and HSV-LIF were PCR positive for HSV DNA, but replicating virus was no longer found from these sections. In addition, there was no general trend of increase of ventricle size. The apparent ventricle size was dependent on section level and location. HE-stainings of all animals were analyzed and typical examples are shown. Scale bars are shown in the figure.
(TIF)

Figure S5 **Clinical scores of EAE with or without treatment with HSV vectors.** The disease score for each mouse was recorded daily based on the following classification: 0 = healthy; 1 = fur ruffling; 2 = tail atonia; 3 = hind limb paralysis and 4 = tetraparalysis. The black squares indicate EAE without virus treatment, the grey squares indicate UV-irradiated vector treated EAE, the orange circles indicate treatment with HSV-Zeo and the red circles indicate treatment with the HSV-LIF. * p<0.05, and ** p<0.01 indicate statistically significant difference when compared to untreated EAE mice on indicated days. # indicates significant difference when compared to UV-irradiated vector treated mice (p<0.05) on indicated days. Data is presented as mean ± SEM.
(TIF)

Figure S6 **Histopathological changes (H & E) with inflammatory cell infiltrations in brain.** Inflammatory infiltrates were found adjacent to the ventricles in brains of untreated (A), UV-irradiated vector (B), HSV-Zeo (C) and HSV-LIF treated EAE mice (D). Squares indicate areas shown at higher magnification. Scale bars are as indicated in the pictures. (E) Severity of inflammation was scored from CNS samples. No significant differences were observed between the groups. The scoring was: 0 = no infiltration; 1 = perivascular infiltration; 2 = perivascular inflammatory cuffs; 3 = inflammation of the brain substance. Each group at each time point consisted of five mice.
(TIF)

Figure S7 **Histopathological changes (H & E) with inflammatory cell infiltrations in spinal cord.** Inflammatory infiltrates were detected in spinal cords of untreated (A), UV-irradiated vector (B), HSV-Zeo (C) and HSV-LIF treated EAE mice (D). Squares indicate areas shown at higher magnification. Scale bars are as indicated in the pictures. (E) Severity of inflammation was scored from CNS samples. No significant differences were observed between the groups. The scoring was: 0 = no infiltration; 1 = perivascular infiltration; 2 = perivascular inflammatory cuffs; 3 = inflammation of the brain substance. Each group at each time point consisted of five mice.
(TIF)

Figure S8 **mRNA expression of T cell population markers in brains and spinal cords of HSV vector-treated and untreated EAE mice.** Q-RT-PCR analysis of FoxP3, RORγT, T-bet and GATA3 mRNA expression as relative values adjusted to the housekeeping genes GAPDH and β-actin. Black bar indicates EAE without treatment, dark grey bar UV-irradiated vector treatment, orange bar treatment with HSV-Zeo and red bar treatment with HSV-LIF. * and ** indicate significant changes (p<0.05 and p<0.01, respectively) in comparison to untreated mice at different time points. Five mice per group were included at each time point. The relative copy number values are shown on a logarithmic scale. SDs are shown.
(TIF)

Table S1 **Primers used for construction of HSV BACs and for quantitative real-time PCR.**
(DOC)

Table S2 **PCR detection of transgene and viral DNA in the nervous system in the different treatment groups.**
(DOC)

Materials and Methods S1 (DOC)

## References

[1] Frampton AJ, Goins W, Nakano K, Burton E, Glorioso J (2005) HSV trafficking and development of gene therapy vectors with applications in the nervous system. Gene Ther 12: 891–901. 15908995 10.1038/sj.gt.3302545

[2] Manservigi R, Argnani R, Marconi P (2010) HSV Recombinant Vectors for Gene Therapy. Open Virol J 4: 123–156. 20835362 10.2174/1874357901004010123 PMC2936037

[3] Chou J, Kern ER, Whitley RJ, Roizman B (1990) Mapping of herpes simplex virus-1 neurovirulence to gamma 134.5, a gene nonessential for growth in culture. Science 250: 1262–1266. 2173860 10.1126/science.2173860

[4] Chou J, Roizman B (1992) The gamma 1(34.5) gene of herpes simplex virus 1 precludes neuroblastoma cells from triggering total shutoff of protein synthesis characteristic of programed cell death in neuronal cells. Proc Natl Acad Sci U S A 89: 3266–3270. 1314384 10.1073/pnas.89.8.3266 PMC48847

[5] Whitley R, Kern E, Chatterjee S, Chou J, Roizman B (1993) Replication, establishment of latency, and induced reactivation of herpes simplex virus gamma 1 34.5 deletion mutants in rodent models. J Clin Invest 91: 2837–2843. 8390490 10.1172/JCI116527 PMC443352

[6] Markovitz N, Baunoch D, Roizman B (1997) The range and distribution of murine central nervous system cells infected with the gamma(1)34.5- mutant of herpes simplex virus 1. J Virol 71: 5560–5569. 9188630 10.1128/jvi.71.7.5560-5569.1997 PMC191798

[7] He B, Gross M, Roizman B (1997) The gamma(1)34.5 protein of herpes simplex virus 1 complexes with protein phosphatase 1alpha to dephosphorylate the alpha subunit of the eukaryotic translation initiation factor 2 and preclude the shutoff of protein synthesis by double-stranded RNA-activated protein kinase. Proc Natl Acad Sci U S A 94: 843–848. 9023344 10.1073/pnas.94.3.843 PMC19601

[8] Verpooten D, Ma Y, Hou S, Yan Z, He B (2009) Control of TANK-binding kinase 1-mediated signaling by the gamma(1)34.5 protein of herpes simplex virus 1. J Biol Chem 284: 1097–1105. 19010780 10.1074/jbc.M805905200 PMC2613634

[9] Andreansky S, He B, van Cott J, McGhee J, Markert J, et al (1998) Treatment of intracranial gliomas in immunocompetent mice using herpes simplex viruses that express murine interleukins. Gene Ther 5: 121–130. 9536273 10.1038/sj.gt.3300550

[10] Martuza RL (2000) Conditionally replicating herpes vectors for cancer therapy. J Clin Invest 105: 841–846. 10749560 10.1172/JCI9744 PMC382984

[11] Campadelli-Fiume G, De Giovanni C, Gatta V, Nanni P, Lollini PL, et al (2011) Rethinking herpes simplex virus: the way to oncolytic agents. Rev Med Virol 21: 213–226. 21626603 10.1002/rmv.691

[12] Furlan R, Poliani P, Galbiati F, Bergami A, Grimaldi L, et al (1998) Central nervous system delivery of interleukin 4 by a nonreplicative herpes simplex type 1 viral vector ameliorates autoimmune demyelination. Hum Gene Ther 9: 2605–2617. 9853527 10.1089/hum.1998.9.17-2605

[13] Broberg E, Setälä N, Röyttä M, Salmi A, Erälinna J, et al (2001) Expression of interleukin-4 but not of interleukin-10 from a replicative herpes simplex virus type 1 viral vector precludes experimental allergic encephalomyelitis. Gene Ther 8: 769–777. 11420640 10.1038/sj.gt.3301465

[14] Nygårdas M, Aspelin C, Paavilainen H, Röyttä M, Waris M, et al (2011) Treatment of experimental autoimmune encephalomyelitis in SJL/J mice with a replicative HSV-1 vector expressing interleukin-5. Gene Ther 18: 646–655. 21326329 10.1038/gt.2011.4

[15] Noseworthy JH, Lucchinetti C, Rodriguez M, Weinshenker BG (2000) Multiple sclerosis. N Engl J Med 343: 938–952. 11006371 10.1056/NEJM200009283431307

[16] Zamvil S, Steinman L (1990) The T lymphocyte in experimental allergic encephalomyelitis. Annu Rev Immunol 8: 579–621. 2188675 10.1146/annurev.iy.08.040190.003051

[17] Langrish C, Chen Y, Blumenschein W, Mattson J, Basham B, et al (2005) IL-23 drives a pathogenic T cell population that induces autoimmune inflammation. J Exp Med 201: 233–240. 15657292 10.1084/jem.20041257 PMC2212798

[18] Kennedy M, Torrance D, Picha K, Mohler K (1992) Analysis of cytokine mRNA expression in the central nervous system of mice with experimental autoimmune encephalomyelitis reveals that IL-10 mRNA expression correlates with recovery. J Immunol 149: 2496–2505. 1527389

[19] Cua DJ, Hinton DR, Stohlman SA (1995) Self-antigen-induced Th2 responses in experimental allergic encephalomyelitis (EAE)-resistant mice. Th2-mediated suppression of autoimmune disease. J Immunol 155: 4052–4059. 7561116

[20] O'Connor RA, Anderton SM (2008) Foxp3+ regulatory T cells in the control of experimental CNS autoimmune disease. J Neuroimmunol 193: 1–11. 18077005 10.1016/j.jneuroim.2007.11.016

[21] Sakaguchi S (2004) Naturally arising CD4+ regulatory t cells for immunologic self-tolerance and negative control of immune responses. Annu Rev Immunol 22: 531–562. 15032588 10.1146/annurev.immunol.21.120601.141122

[22] Fletcher J, Lalor S, Sweeney C, Tubridy N, Mills K (2010) T cells in multiple sclerosis and experimental autoimmune encephalomyelitis. Clin Exp Immunol 162: 1–11. 20682002 10.1111/j.1365-2249.2010.04143.x PMC2990924

[23] Rudick RA, Stuart WH, Calabresi PA, Confavreux C, Galetta SL, et al (2006) Natalizumab plus interferon beta-1a for relapsing multiple sclerosis. N Engl J Med 354: 911–923. 16510745 10.1056/NEJMoa044396

[24] Polman CH, O'Connor PW, Havrdova E, Hutchinson M, Kappos L, et al (2006) A randomized, placebo-controlled trial of natalizumab for relapsing multiple sclerosis. N Engl J Med 354: 899–910. 16510744 10.1056/NEJMoa044397

[25] Comi G, Filippi M, Wolinsky JS (2001) European/Canadian multicenter, double-blind, randomized, placebo-controlled study of the effects of glatiramer acetate on magnetic resonance imaging–measured disease activity and burden in patients with relapsing multiple sclerosis. European/Canadian Glatiramer Acetate Study Group. Ann Neurol 49: 290–297. 11261502

[26] Kappos L, Freedman MS, Polman CH, Edan G, Hartung HP, et al (2009) Long-term effect of early treatment with interferon beta-1b after a first clinical event suggestive of multiple sclerosis: 5-year active treatment extension of the phase 3 BENEFIT trial. Lancet Neurol 8: 987–997. 19748319 10.1016/S1474-4422(09)70237-6

[27] Jacobs LD, Cookfair DL, Rudick RA, Herndon RM, Richert JR, et al (1996) Intramuscular interferon beta-1a for disease progression in relapsing multiple sclerosis. The Multiple Sclerosis Collaborative Research Group (MSCRG). Ann Neurol 39: 285–294. 8602746 10.1002/ana.410390304

[28] Gearing DP, Thut CJ, VandeBos T, Gimpel SD, Delaney PB, et al (1991) Leukemia inhibitory factor receptor is structurally related to the IL-6 signal transducer, gp130. EMBO J 10: 2839–2848. 1915266 10.1002/j.1460-2075.1991.tb07833.x PMC452994

[29] Gearing DP, Comeau MR, Friend DJ, Gimpel SD, Thut CJ, et al (1992) The IL-6 signal transducer, gp130: an oncostatin M receptor and affinity converter for the LIF receptor. Science 255: 1434–1437. 1542794 10.1126/science.1542794

[30] Metcalf D (2003) The unsolved enigmas of leukemia inhibitory factor. Stem Cells 21: 5–14. 12529546 10.1634/stemcells.21-1-5

[31] Brown MA, Metcalf D, Gough NM (1994) Leukaemia inhibitory factor and interleukin 6 are expressed at very low levels in the normal adult mouse and are induced by inflammation. Cytokine 6: 300–309. 8054487 10.1016/1043-4666(94)90027-2

[32] Kerr BJ, Patterson PH (2005) Leukemia inhibitory factor promotes oligodendrocyte survival after spinal cord injury. Glia 51: 73–79. 15779090 10.1002/glia.20177

[33] Azari MF, Profyris C, Karnezis T, Bernard CC, Small DH, et al (2006) Leukemia inhibitory factor arrests oligodendrocyte death and demyelination in spinal cord injury. J Neuropathol Exp Neurol 65: 914–929. 16957585 10.1097/01.jnen.0000235855.77716.25

[34] Slaets H, Dumont D, Vanderlocht J, Noben JP, Leprince P, et al (2008) Leukemia inhibitory factor induces an antiapoptotic response in oligodendrocytes through Akt-phosphorylation and up-regulation of 14-3-3. Proteomics 8: 1237–1247. 18338825 10.1002/pmic.200700641

[35] Sugiura S, Lahav R, Han J, Kou SY, Banner LR, et al (2000) Leukaemia inhibitory factor is required for normal inflammatory responses to injury in the peripheral and central nervous systems in vivo and is chemotactic for macrophages in vitro. Eur J Neurosci 12: 457–466. 10712626 10.1046/j.1460-9568.2000.00922.x

[36] Mahic M, Kalland ME, Aandahl EM, Torgersen KM, Taskén K (2008) Human naturally occurring and adaptive regulatory T cells secrete high levels of leukaemia inhibitory factor upon activation. Scand J Immunol 68: 391–396. 18782268 10.1111/j.1365-3083.2008.02148.x

[37] Gao W, Thompson L, Zhou Q, Putheti P, Fahmy TM, et al (2009) Treg versus Th17 lymphocyte lineages are cross-regulated by LIF versus IL-6. Cell Cycle 8: 1444–1450. 19342884 10.4161/cc.8.9.8348 PMC2881570

[38] Vanderlocht J, Hellings N, Hendriks JJ, Vandenabeele F, Moreels M, et al (2006) Leukemia inhibitory factor is produced by myelin-reactive T cells from multiple sclerosis patients and protects against tumor necrosis factor-alpha-induced oligodendrocyte apoptosis. J Neurosci Res 83: 763–774. 16477612 10.1002/jnr.20781

[39] Butzkueven H, Zhang J, Soilu-Hanninen M, Hochrein H, Chionh F, et al (2002) LIF receptor signaling limits immune-mediated demyelination by enhancing oligodendrocyte survival. Nat Med 8: 613–619. 12042813 10.1038/nm0602-613

[40] Butzkueven H, Emery B, Cipriani T, Marriott M, Kilpatrick T (2006) Endogenous leukemia inhibitory factor production limits autoimmune demyelination and oligodendrocyte loss. Glia 53: 696–703. 16498619 10.1002/glia.20321

[41] Gresle MM, Alexandrou E, Wu Q, Egan G, Jokubaitis V, et al (2012) Leukemia Inhibitory Factor Protects Axons in Experimental Autoimmune Encephalomyelitis via an Oligodendrocyte-Independent Mechanism. PLoS One 7: e47379. 23077604 10.1371/journal.pone.0047379 PMC3471848

[42] Slaets H, Hendriks JJ, Van den Haute C, Coun F, Baekelandt V, et al (2010) CNS-targeted LIF expression improves therapeutic efficacy and limits autoimmune-mediated demyelination in a model of multiple sclerosis. Mol Ther 18: 684–691. 20068552 10.1038/mt.2009.311 PMC2862528

[43] Messerle M, Crnkovic I, Hammerschmidt W, Ziegler H, Koszinowski UH (1997) Cloning and mutagenesis of a herpesvirus genome as an infectious bacterial artificial chromosome. Proc Natl Acad Sci U S A 94: 14759–14763. 9405686 10.1073/pnas.94.26.14759 PMC25110

[44] Adler H, Messerle M, Koszinowski UH (2003) Cloning of herpesviral genomes as bacterial artificial chromosomes. Rev Med Virol 13: 111–121. 12627394 10.1002/rmv.380

[45] Nagel CH, Döhner K, Fathollahy M, Strive T, Borst EM, et al (2008) Nuclear egress and envelopment of herpes simplex virus capsids analyzed with dual-color fluorescence HSV1(17+). J Virol 82: 3109–3124. 18160444 10.1128/JVI.02124-07 PMC2258981

[46] Sandbaumhüter M, Döhner K, Schipke J, Binz A, Pohlmann A, et al.. (2012) Cytosolic herpes simplex virus capsids not only require binding inner tegument protein pUL36 but also pUL37 for active transport prior to secondary envelopment. Cell Microbiol doi: 10.1111/cmi.12075. 10.1111/cmi.12075 23186167

[47] Abramoff MD, Magelhaes PJ, Ram SJ (2004) Image Processing with ImageJ. Biophotonics International 11: 36–42.

[48] Broberg E, Setälä N, Erälinna J, Salmi A, Röyttä M, et al (2002) Herpes simplex virus type 1 infection induces upregulation of interleukin-23 (p19) mRNA expression in trigeminal ganglia of BALB/c mice. J Interferon Cytokine Res 22: 641–651. 12162874 10.1089/10799900260100123

[49] Hukkanen V, Rehn T, Kajander R, Sjöroos M, Waris M (2000) Time-resolved fluorometry PCR assay for rapid detection of herpes simplex virus in cerebrospinal fluid. J Clin Microbiol 38: 3214–3218. 10970360 10.1128/jcm.38.9.3214-3218.2000 PMC87359

[50] Berges BK, Wolfe JH, Fraser NW (2007) Transduction of brain by herpes simplex virus vectors. Mol Ther 15: 20–29. 17164771 10.1038/sj.mt.6300018

[51] Martino G, Furlan R, Comi G, Adorini L (2001) The ependymal route to the CNS: an emerging gene-therapy approach for MS. Trends Immunol 22: 483–490. 11525938 10.1016/s1471-4906(01)01990-1

[52] Kesari S, Lasner TM, Balsara KR, Randazzo BP, Lee VM, et al (1998) A neuroattenuated ICP34.5-deficient herpes simplex virus type 1 replicates in ependymal cells of the murine central nervous system. J Gen Virol 79 (Pt 3): 525–536. 10.1099/0022-1317-79-3-525 9519831

[53] Gierasch WW, Zimmerman DL, Ward SL, Vanheyningen TK, Romine JD, et al (2006) Construction and characterization of bacterial artificial chromosomes containing HSV-1 strains 17 and KOS. J Virol Methods 135: 197–206. 16647145 10.1016/j.jviromet.2006.03.014

[54] Baker D, Gerritsen W, Rundle J, Amor S (2011) Critical appraisal of animal models of multiple sclerosis. Mult Scler 17: 647–657. 21372117 10.1177/1352458511398885

[55] Banner LR, Moayeri NN, Patterson PH (1997) Leukemia inhibitory factor is expressed in astrocytes following cortical brain injury. Exp Neurol 147: 1–9. 9294397 10.1006/exnr.1997.6536

[56] Yoshida T, Satoh M, Nakagaito Y, Kuno H, Takeuchi M (1993) Cytokines affecting survival and differentiation of an astrocyte progenitor cell line. Brain Res Dev Brain Res 76: 147–150. 8306427 10.1016/0165-3806(93)90132-t

[57] Cantin E, Hinton D, Chen J, Openshaw H (1995) Gamma interferon expression during acute and latent nervous system infection by herpes simplex virus type 1. J Virol 69: 4898–4905. 7609058 10.1128/jvi.69.8.4898-4905.1995 PMC189304

[58] Guo B, Chang E, Cheng G (2008) The type I IFN induction pathway constrains Th17-mediated autoimmune inflammation in mice. J Clin Invest 118: 1680–1690. 18382764 10.1172/JCI33342 PMC2276397

[59] Prinz M, Schmidt H, Mildner A, Knobeloch K, Hanisch U, et al (2008) Distinct and nonredundant in vivo functions of IFNAR on myeloid cells limit autoimmunity in the central nervous system. Immunity 28: 675–686. 18424188 10.1016/j.immuni.2008.03.011

