# Supplementary figures and images for: A Herpes Simplex Virus-Derived Replicative Vector Expressing LIF Limits Experimental Demyelinating Disease and Modulates Autoimmunity
Source: PLoS One. 2013 May 20;8(5):e64200. doi: 10.1371/journal.pone.0064200 (PMC3659099; doi:10.1371/journal.pone.0064200)

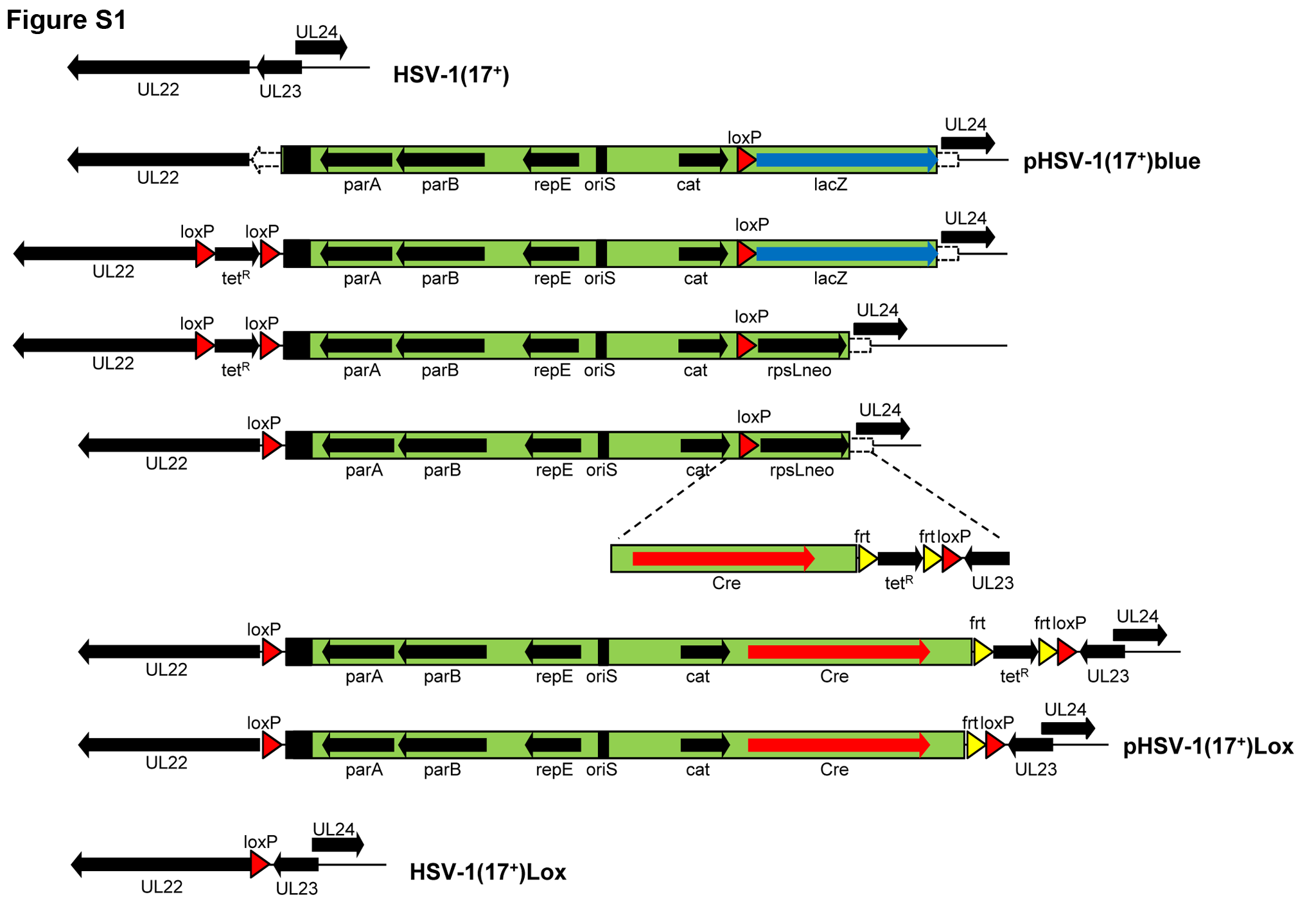

Supplement: Figure S1 — Repair of UL23 in the HSV-1(17+) BAC. Schematic representation of the mutagenesis procedure for the repair of UL23 gene and the insertion of a Cre/Lox recombination system into pHSV-1(17+)blue [45]. After transfection of eukaryotic cells with the resulting BAC pHSV-1(17+)Lox, Cre recombinase excises the non-viral sequences (depicted in the green box) from the genome, leaving a single loxP site between the UL22 and UL23 ORFs. The gene structure of the parental virus is shown on the top line and the resulting BAC-derived virus genome is shown at the bottom.Abbreviations: parA-B, repE, oriS: BAC replication origin and regulatory genes. cat: chloramphenicol resistance, tetR: tetracyclin resistance, lacZ: eukaryotic beta-galactosidase expression cassette, loxP: Cre recombinase recognition site, rpsLneo: streptomycin sensitivity/kanamycin resistance counterselection/selection cassette, Cre: eukaryotic Cre recombinase expression cassette. frt: Flp recombinase recognition site. (TIF) [file pone.0064200.s001.tif]

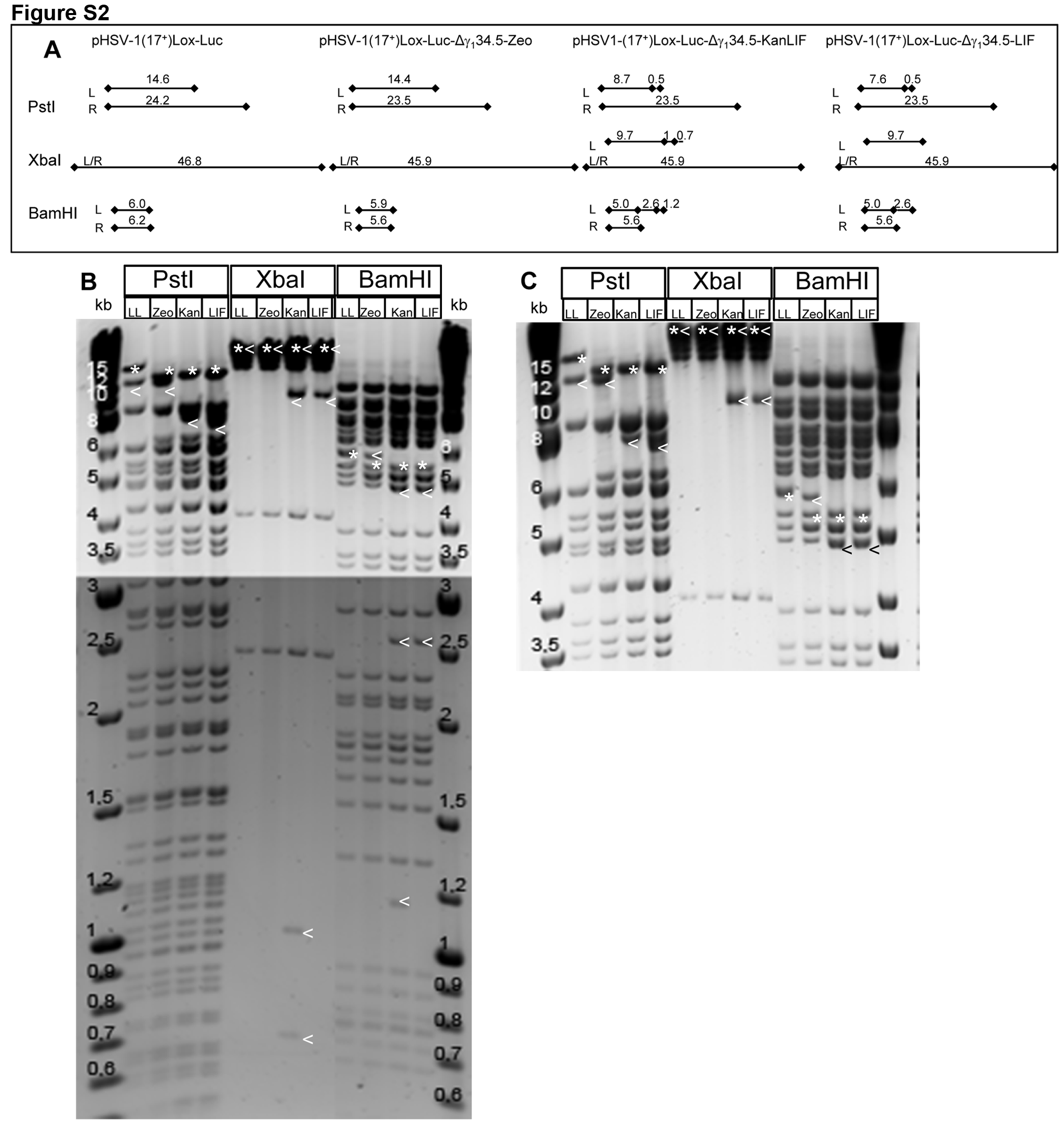

Supplement: Figure S2 — Restriction endonuclease patterns of HSV BAC DNA. Restriction profiles of chosen BAC clones of pHSV-1(17+)Lox-Luc, pHSV-1(17+)Lox-Luc-Δγ134.5-Zeo, pHSV-1(17+)Lox-Luc-Δγ134.5-KanLIF and pHSV-1(17+)Lox-Luc-Δγ134.5-LIF. (A) Changes in fragment size (kbp) after PstI, XbaI and BamHI restriction digest. L = band shifts in the 5′ area of γ134.5, R = band shifts in the 3′ area of γ134.5, L/R band shifts covering both γ134.5 copies. (B-C) Gel elecrophoresis. (LL) = pHSV-1(17+)Lox-Luc, (Zeo) = pHSV-1(17+)Lox-Luc-Δγ134.5-Zeo, (Kan) = pHSV-1(17+)Lox-Luc-Δγ134.5-KanLIF, (LIF) = pHSV-1(17+)Lox-Luc-Δγ134.5-LIF. Stars (*) indicate changes in restriction pattern after the deletion of the 3′ γ134.5, (<) indicate changes in restriction pattern after modification of the 5′ γ134.5 of HSV-1. Abbreviations: HSV, herpes simplex virus; Zeo, zeocin resistance cassette; LIF, leukemia inhibitory factor. (TIF) [file pone.0064200.s002.tif]

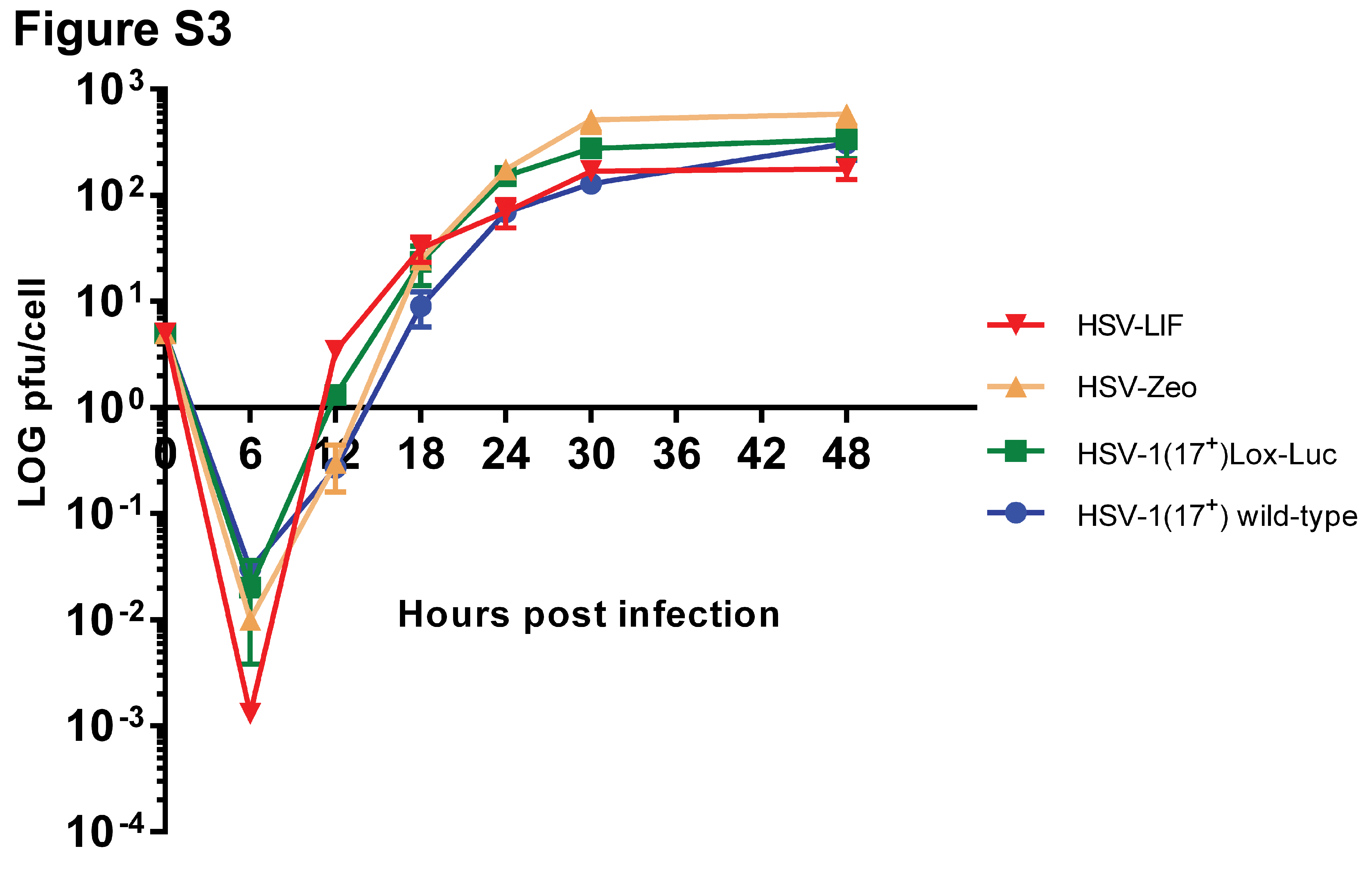

Supplement: Figure S3 — Growth curves of HSV-1(17+) wild-type and BAC-derived HSV vectors. Vero cells (in 6 well-plates) were infected with 5 MOI of respective viruses. Cells were washed at 6 hours post infection to remove the original virus stock. Supernatant samples were taken every six hours. The virus amount in the supernatant was determined with plaque titration. (TIF) [file pone.0064200.s003.tif]

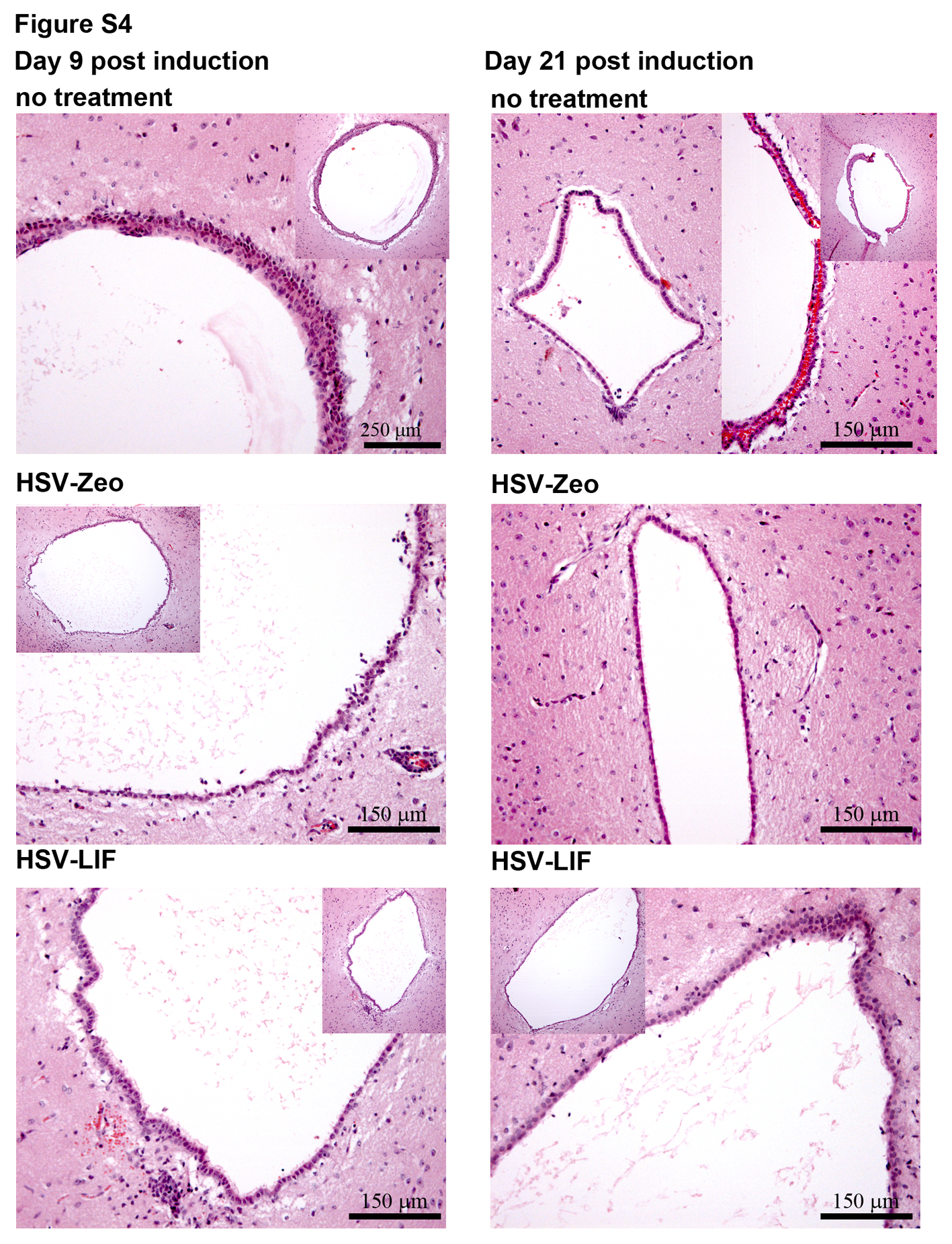

Supplement: Figure S4 — Brain ventricular ependymal cells in mice infected with the γ134.5-deleted HSV vectors. Ependymal cells are shown at the 3rd ventricles in HSV-Zeo and HSV-LIF treated EAE mice. Untreated EAE mice were included as controls. The sections in the left column are parallel to sections positive for HSV in immunohistochemical stainings (Figure 3), representing day 9 post induction (day 3 post infection). The right side panel shows brains representing day 21 post induction (day 15 post infection). No clear difference in ependymal cell line was detected between the treatment groups. The day 21 sections shown for HSV-Zeo and HSV-LIF were PCR positive for HSV DNA, but replicating virus was no longer found from these sections. In addition, there was no general trend of increase of ventricle size. The apparent ventricle size was dependent on section level and location. HE-stainings of all animals were analyzed and typical examples are shown. Scale bars are shown in the figure. (TIF) [file pone.0064200.s004.tif]

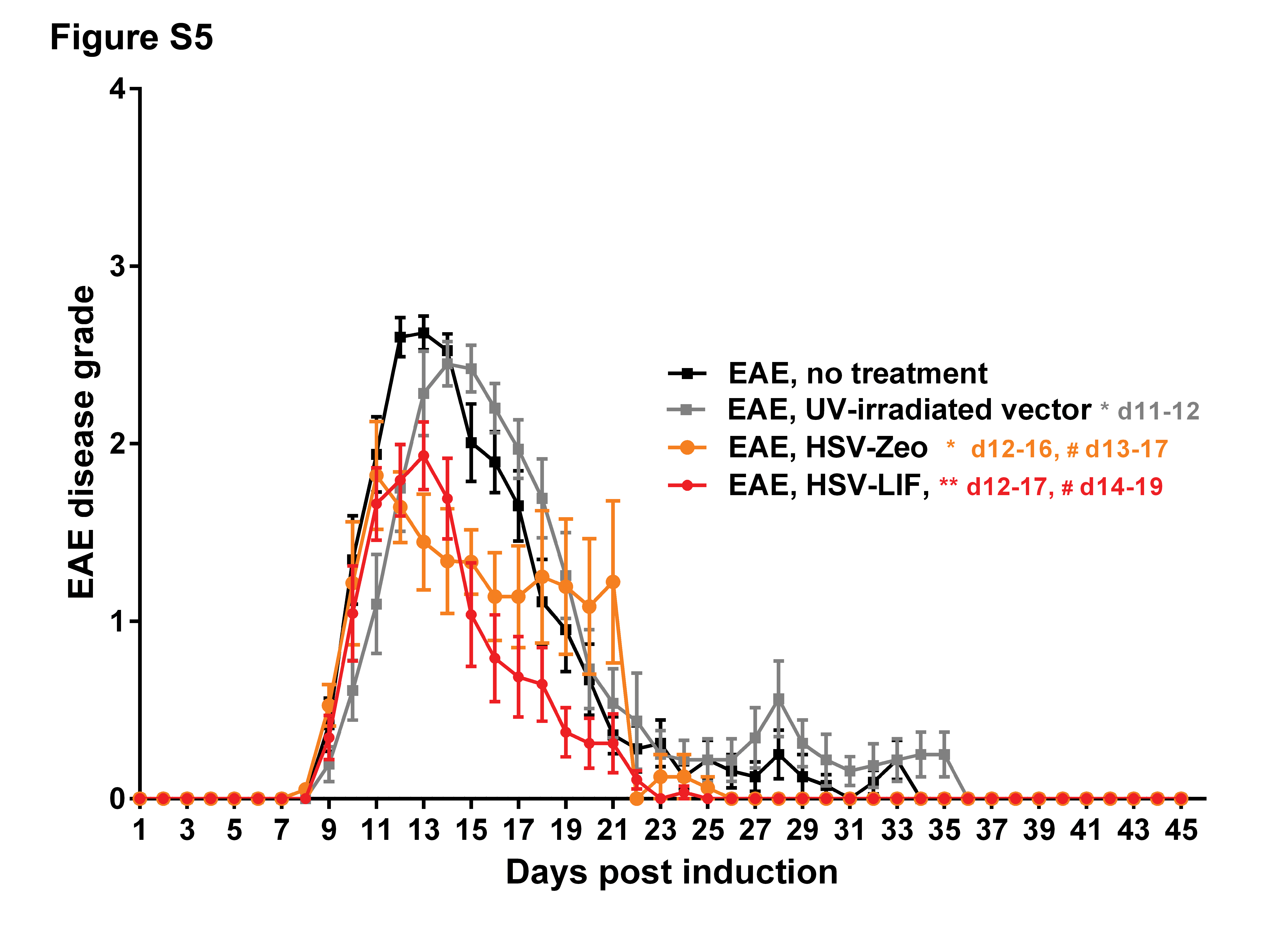

Supplement: Figure S5 — Clinical scores of EAE with or without treatment with HSV vectors. The disease score for each mouse was recorded daily based on the following classification: 0 = healthy; 1 = fur ruffling; 2 = tail atonia; 3 = hind limb paralysis and 4 = tetraparalysis. The black squares indicate EAE without virus treatment, the grey squares indicate UV-irradiated vector treated EAE, the orange circles indicate treatment with HSV-Zeo and the red circles indicate treatment with the HSV-LIF. * p<0.05, and ** p<0.01 indicate statistically significant difference when compared to untreated EAE mice on indicated days. # indicates significant difference when compared to UV-irradiated vector treated mice (p<0.05) on indicated days. Data is presented as mean ± SEM. (TIF) [file pone.0064200.s005.tif]

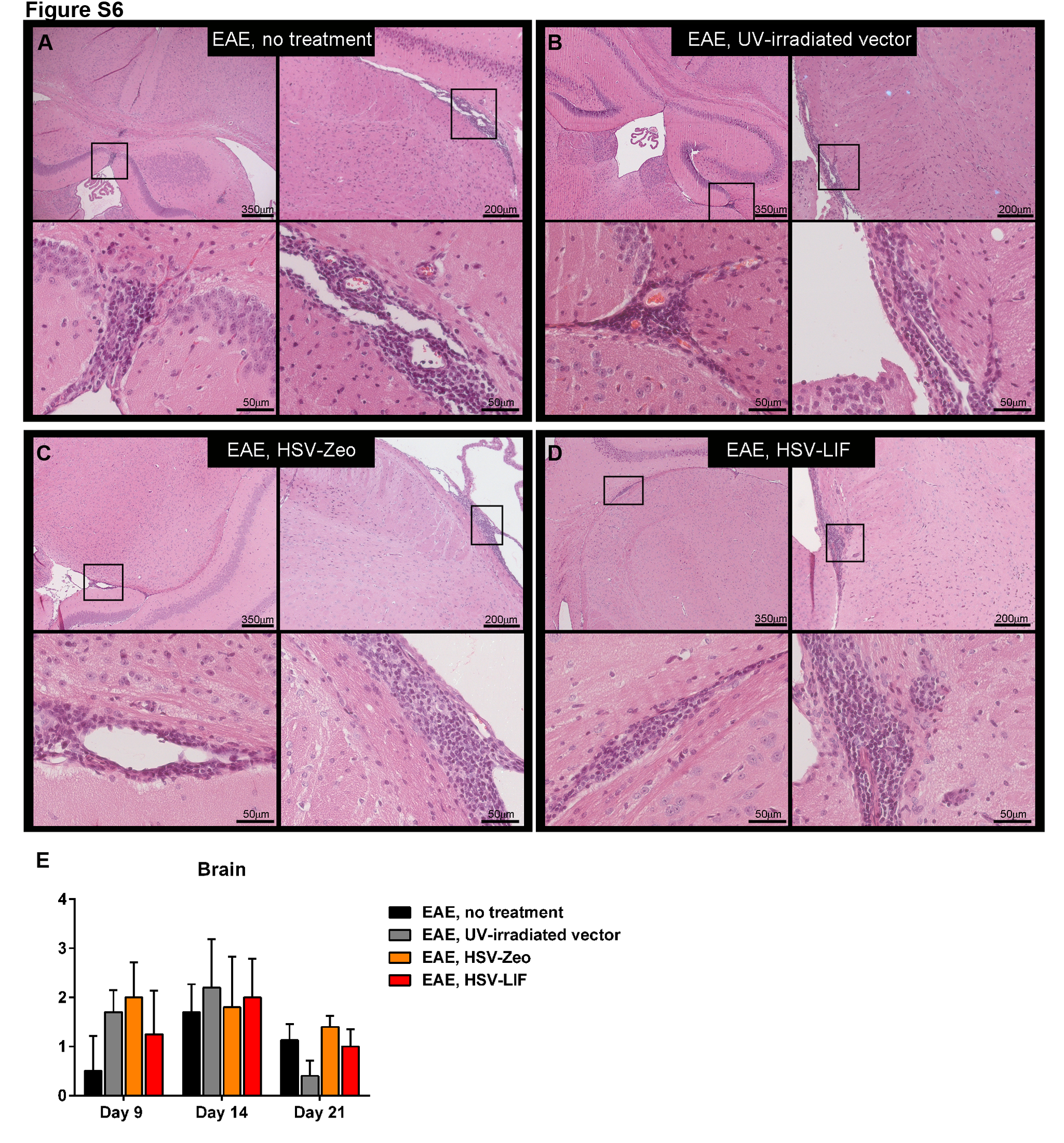

Supplement: Figure S6 — Histopathological changes (H & E) with inflammatory cell infiltrations in brain. Inflammatory infiltrates were found adjacent to the ventricles in brains of untreated (A), UV-irradiated vector (B), HSV-Zeo (C) and HSV-LIF treated EAE mice (D). Squares indicate areas shown at higher magnification. Scale bars are as indicated in the pictures. (E) Severity of inflammation was scored from CNS samples. No significant differences were observed between the groups. The scoring was: 0 = no infiltration; 1 = perivascular infiltration; 2 = perivascular inflammatory cuffs; 3 = inflammation of the brain substance. Each group at each time point consisted of five mice. (TIF) [file pone.0064200.s006.tif]

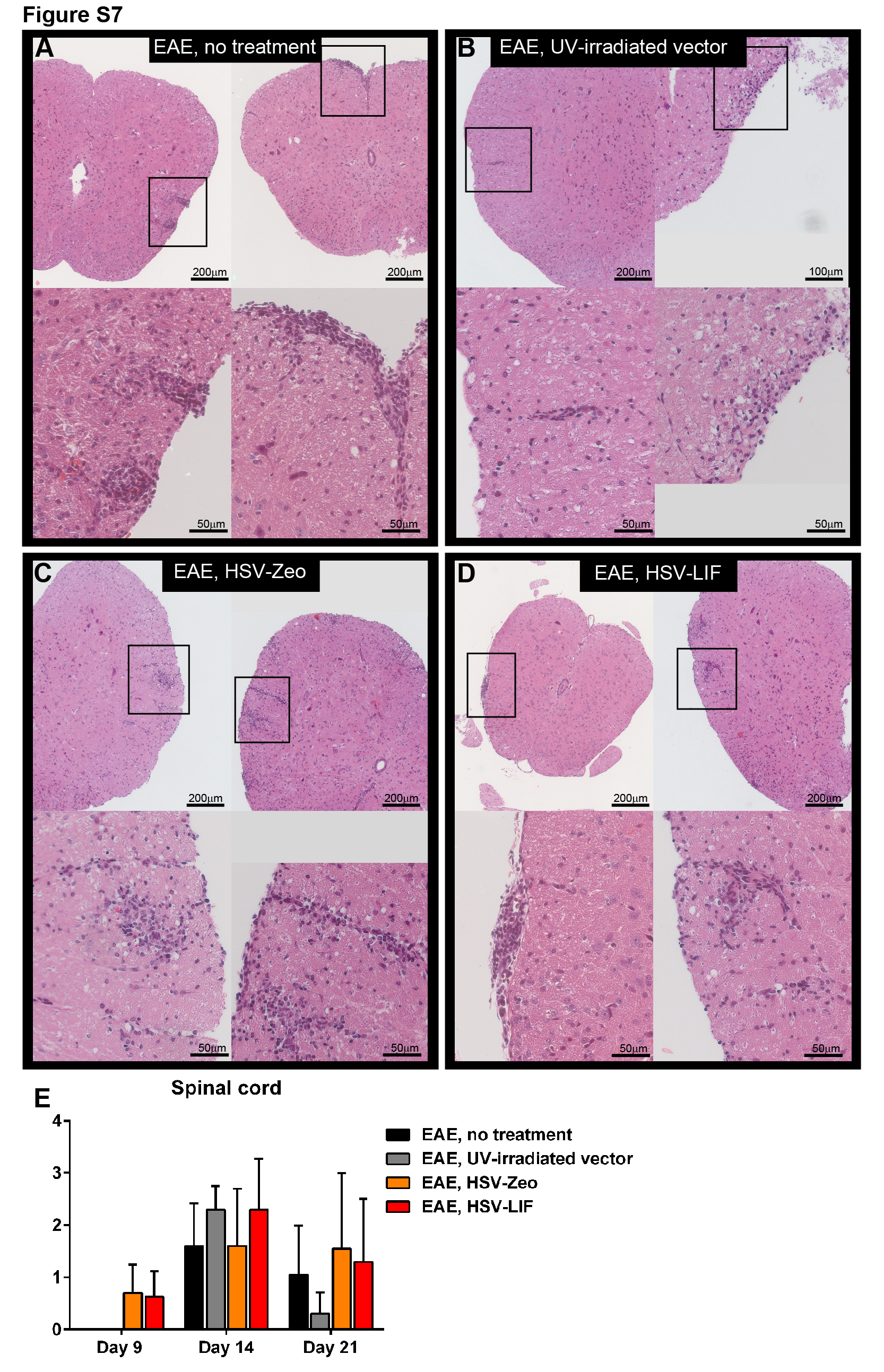

Supplement: Figure S7 — Histopathological changes (H & E) with inflammatory cell infiltrations in spinal cord. Inflammatory infiltrates were detected in spinal cords of untreated (A), UV-irradiated vector (B), HSV-Zeo (C) and HSV-LIF treated EAE mice (D). Squares indicate areas shown at higher magnification. Scale bars are as indicated in the pictures. (E) Severity of inflammation was scored from CNS samples. No significant differences were observed between the groups. The scoring was: 0 = no infiltration; 1 = perivascular infiltration; 2 = perivascular inflammatory cuffs; 3 = inflammation of the brain substance. Each group at each time point consisted of five mice. (TIF) [file pone.0064200.s007.tif]

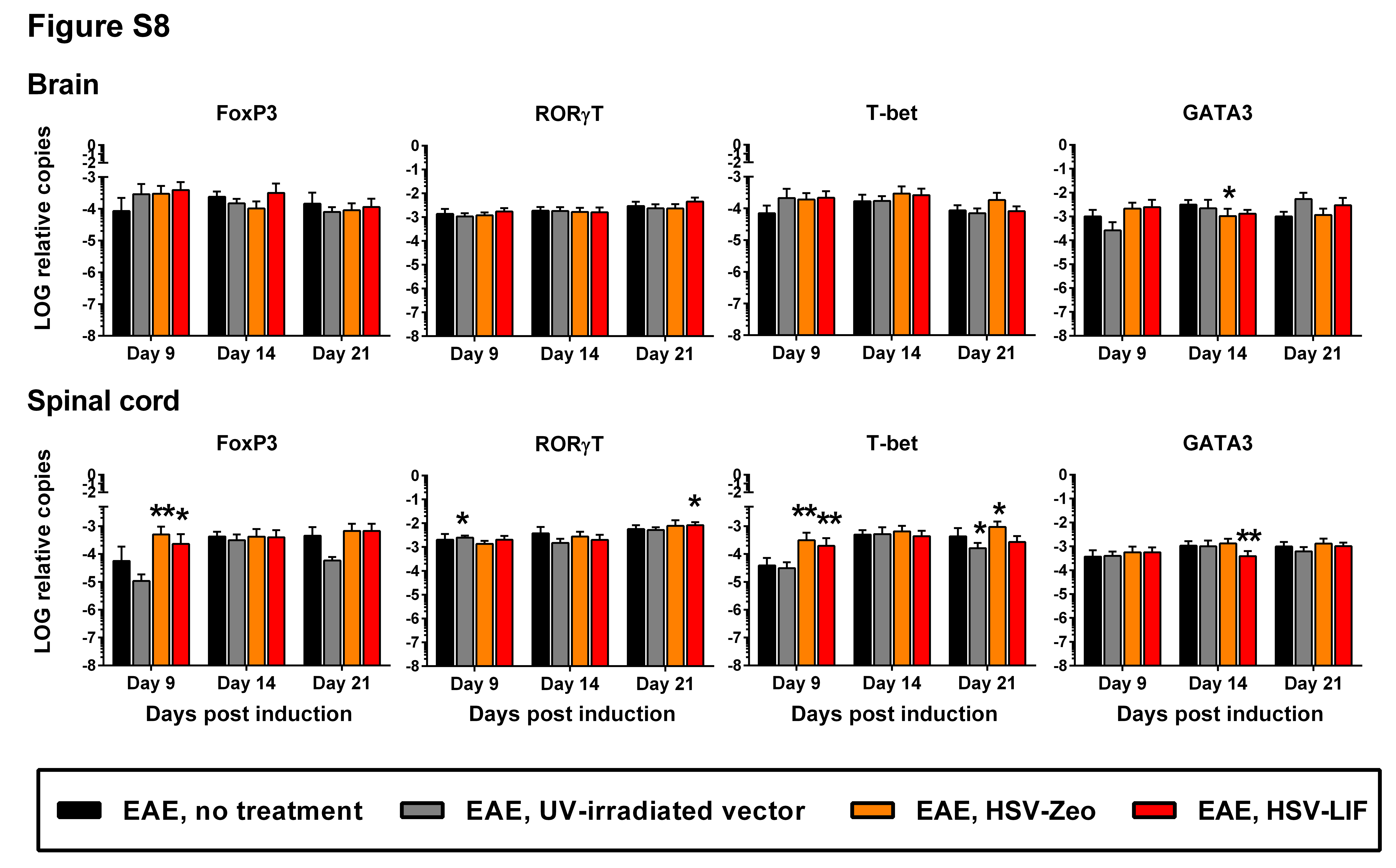

Supplement: Figure S8 — mRNA expression of T cell population markers in brains and spinal cords of HSV vector-treated and untreated EAE mice. Q-RT-PCR analysis of FoxP3, RORγT, T-bet and GATA3 mRNA expression as relative values adjusted to the housekeeping genes GAPDH and β-actin. Black bar indicates EAE without treatment, dark grey bar UV-irradiated vector treatment, orange bar treatment with HSV-Zeo and red bar treatment with HSV-LIF. * and ** indicate significant changes (p<0.05 and p<0.01, respectively) in comparison to untreated mice at different time points. Five mice per group were included at each time point. The relative copy number values are shown on a logarithmic scale. SDs are shown. (TIF) [file pone.0064200.s008.tif]
