# Supplementary material for: A Herpes Simplex Virus-Derived Replicative Vector Expressing LIF Limits Experimental Demyelinating Disease and Modulates Autoimmunity
Source: PLoS One. 2013 May 20;8(5):e64200. doi: 10.1371/journal.pone.0064200 (PMC3659099; doi:10.1371/journal.pone.0064200)
Supplement: Table S1 — Primers used for construction of HSV BACs and for quantitative real-time PCR. (DOC) [file pone.0064200.s009.doc]

**Table S1. Primers used for construction of HSV BACs and for quantitative real-time** PCR.

|  |  |
| --- | --- |
| **Oligonucleotides** | **Sequence (5´- 3´)** |
|  |  |
|  |  |
| **Construction of BACs** ^a^ |  |
| MG011.for | ctg ctg gtg gcc ctg ggt tcg cgc gac gat atc gtc tac *gtt cag aag aac tcg tca aga agg* |
| MG002.rev | gat gaa tgg cag aaa ttc gat gat aag ctg tca aac atg aga att *ggc ctg gtg atg atg gcg gga tc* |
|  |  |
| nm0003-1 | tgt gag tta ata aat aaa agt atc acg gtc cat act ggc ctg tcg cgt tgt ctc tga *cta caa gga cga cga cga ca* |
| nm0002-1 | gag ccg taa ccc aac caa acc agg cgt ggt gtg agt ttg tgg *cat gcc tgc tat tgt ctt ccc a* |
|  |  |
| ICP34.5.for | atg gcc cgc cgc cgc cgc cat cgc ggc ccc *gaa aag tgc cac ctg cag at* |
| ICP34.5.rev | tta gac cga gtt cgc cgg gcc ggc tcc gcg *cag gaa cac tta acg gct ga* |
|  |  |
| ICP34.5zeo.for | gtc cca acc gca cag tcc cag gtc acc *tgt tga caa tta atc atc ggc at* |
| ICP34.5zeo.rev | tta gac cga gtt cgc cgg gcc ggc tcc gcg *tca gtc ctg ctc ctc ggc ca* |
|  |  |
| mLIF ATG-L | agc tgg tac ccc cat aat gaa ggt ctt ggc cgc agg gat tgt gcc ctt act gct gct ggt tct gca ctg gaa aca cgg ggc a*gg gag ccc tct tcc cat cac* |
|  |  |
| mLIF-R | agct agct agct agct aag ctt *cta gaa ggc ctg gac cac c* |
|  |  |
| LIF-KanL2 | tag cgg atc cga gct cgg tac ccc cat aat gaa ggt ctt ggc cgc agg gat tgt gcc ctt ata ggg ata aca ggg taa tcg att t*ag gac gac gac gac aag taa* |
|  |  |
| LIF-KanR | agc tag ctg gat cc*c agg aac act taa cgg ctg a* |
|  |  |
| LIF-ICP34.5-L | ccc ggg ccc acg ggc gcc gtc cca acc gca cag tcc cag gta acc *aat tcg gag tgc ctc gtg ag* |
| LIF-ICP34.5-R | agg ccg cct cgg gtg taa cgt tag acc gag ttc gcc ggg ccg gct ccg cg *ctg gtt ctt tcc gcc tca g* |
|  |  |
|  |  |
| **Quantitative RT-PCR** |  |
| mLIF s | aac ggg aca gag aag acc aa |
| mLIF a | cca cac ggt act tgt tgc ac |
|  |  |
| EF1alpha-LIF f (p-LIF f) | tgg aat ttg ccc ttt ttg ag |
| EF1alpha-LIF r (p-LIF r) | tga gct gtg cca gtt gat tc |
|  |  |
| Luc-s | tgt cgc tct gcc tca tag aa |
| Luc-a | aaa ccg tga tgg aat gga ac |
|  |  |
| mGAPDH s | gca agg tca tcc cag agc |
| mGAPDH a | ggt cct cag tgt agc cca ag |
|  |  |
| mFoxP3 s | ctg cat cgt agc cac cag ta |
| mFoxP3 a | gtg gaa gaa ctc tgg gaa gg |
|  |  |
| mIL6 s | gat gga tgc tac caa act gga |
| mIL6 a | ctg aag gac tct ggc ttt gtc |
|  |  |
| mRoRγT s | aa ggc aaa tac ggt ggt gtg |
| mRoRγT a | agg gca atc tca tcc tca ga |
|  |  |
| mTGFβ s | tgc ttc agc tcc aca gag aa |
| mTGFβ a | cag aag ttg gca tgg tag cc |
|  |  |
| mGATA3 s | aag gca ggg agt gtg tga ac |
| mGATA3 a | ctt cgc ttg ggc ttg ata a |
|  |  |
| mT-bet s | gtt ccc agc cgt ttc tac c |
| mT-bet a | tag gag tgt ggg ctt cat gc |
|  |  |
| mSTAT3 s | tct cct tct ggg tct ggc ta |
| mSTAT3 a | cgc tcc ttg ctg atg aaa c |

^a^ Cloning primers: Sequence annealing to template is highlighted in italics.
