# Supplementary material for: A Herpes Simplex Virus-Derived Replicative Vector Expressing LIF Limits Experimental Demyelinating Disease and Modulates Autoimmunity
Source: PLoS One. 2013 May 20;8(5):e64200. doi: 10.1371/journal.pone.0064200 (PMC3659099; doi:10.1371/journal.pone.0064200)
Supplement: Table S2 — PCR detection of transgene and viral DNA in the nervous system in the different treatment groups. (DOC) [file pone.0064200.s010.doc]

**Table S2. PCR detection of transgene and viral DNA in the nervous system in the different treatment groups.**

| **Brain** | Day 9 p.induction  Day 3 p.infection | Day 14 p. induction  Day 8 p.infection | Day 21 p.induction  Day 15 p. infection |
| --- | --- | --- | --- |
| *PCR target* | gD^a^ p-LIF^b^ | gD p-LIF | gD p-LIF |
| EAE, no treatment | 0 0/5^c^ | 0 0/5 | 0 0/5 |
| EAE, UV-irradiated vector^d^ | 0 0/5 | 0 0/5 | 0 0/5 |
| EAE, HSV-Zeo | 5 0/5 | 3 0/5 | 2 0/5 |
| EAE, HSV-LIF | 4 4/4 | 5 2/5 | 2 2/5 |
|  |  |  |  |
| **Trigeminal ganglion** | Day 9 p.induction  Day 3 p.infection | Day 14 p. induction  Day 8 p.infection | Day 21 p.induction  Day 15 p. infection |
| *PCR target* | gD p-LIF | gD p-LIF | gD p-LIF |
| EAE, no treatment | 0 0/5 | 0 0/5 | 0 0/5 |
| EAE, UV-irradiated vector | 0 0/5 | 0 0/5 | 0 0/5 |
| EAE, HSV-Zeo | 4 0/5 | 1 0/5 | 0 0/5 |
| EAE, HSV-LIF | 3 4/4 | 1 1/5 | 0 0/5 |
|  |  |  |  |
| **Spinal cord** | Day 9 p.induction  Day 3 p.infection | Day 14 p. induction  Day 8 p.infection | Day 21 p.induction  Day 15 p. infection |
| *PCR target* | gD p-LIF | gD p-LIF | gD p-LIF |
| EAE, no treatment | 0 0/5 | 0 0/5 | 0 0/5 |
| EAE, UV-irradiated vector | 2 0/5 | 0 0/5 | 0 0/5 |
| EAE, HSV-Zeo | 5 0/5 | 3 0/5 | 2 0/5 |
| EAE, HSV-LIF | 4 2/4 | 3 0/5 | 3 1/5 |

^a^ The HSV-1 gene gD was used as a target for quantitation of viral DNA in brain, spinal cord and trigeminal ganglion samples.

^b^ p-LIF = transgene LIF was verified from the DNA in brain, trigeminal ganglion and spinal cord samples by real-time PCR, annealing to the EF1α promoter and LIF (p-LIF).

^c^ Number of positive mice/number of studied mice.

^d^ UV-inactivated HSV-LIF.
