# Supplementary material for: A Herpes Simplex Virus-Derived Replicative Vector Expressing LIF Limits Experimental Demyelinating Disease and Modulates Autoimmunity
Source: PLoS One. 2013 May 20;8(5):e64200. doi: 10.1371/journal.pone.0064200 (PMC3659099; doi:10.1371/journal.pone.0064200)
Supplement: Materials and Methods S1 — (DOC) [file pone.0064200.s011.doc]

**Materials and Methods S1**

**Construction of recombinant HSV-1(17^+^) BACs.** To generate the bacterial artificial chromosome (BAC) pHSV-1(17^+^) blue (Figure S1), an infectious BAC of the strain HSV-1(17^+^), the genes required for replication in *E. coli* (cat, oriS, repE, parB and parA), a single loxP site and a β-galactosidase expression cassette have been inserted into the UL23 locus (thymidine kinase, TK) [1]. For further mutagenesis, the BACs were grown in the *E. coli* strain DY380, which has the Red recombination system of bacteriophage λ inserted into its genome [2]. *E. coli* DY380 containing the respective BAC were transformed with linear recombination DNA fragments by electroporation and grown at 32°C with the appropriate antibiotics for positive selection. At each mutagenesis step, correct clones were identified based on restriction digest patterns (Figure S2).

To restore thymidine kinase expression and to remove as much foreign sequence as possible, the UL23 locus was repaired and the non-viral sequences were flanked by two loxP sites to allow Cre recombinase-mediated removal of these sequences after reconstitution of HSV-1 in eukaryotic cells (Figure S1). In a first step of homologous recombination and selection we replaced the remaining 168 nt of the 3҆ UL23 ORF (nt 46673-46840; GenBank NC_001806.1) by a loxP sequence-flanked tetracycline resistance. After expression of Cre recombinase from the plasmid pMCre [3], the tetracyclin resistance cassette was excised, leaving one loxP site flanked by the linker sequences AGAGCTCCACCGCGGTGGCGGCCGC and GGATCCAGCTTATCGATACCGTCGAC at the 5' end of the BAC origin (Figure S1). The β-galactosidase expression cassette downstream of the BAC genes was replaced by an rpsL-neo cassette which was amplified from pRpsLneo (GeneBridges, Dresden, Germany) with primers MG011.for and MG002.rev (Table S1). For the next step, a cassette was constructed consisting of 655 bp homologous to the 3' end of the BAC genes, followed by a Cre expression cassette [4], a tetracyclin resistance flanked by FRT sites [5], a single loxP site and the complete UL23 open reading frame (ORF). This cassette was inserted via Red recombination and thereby replaced the original loxP site and the rpsLneo cassette, while restoring the complete UL23 ORF. Afterwards, Flp recombinase mediated the removal of the tetracyclin resistance (pCP20, [6]). The final vector pHSV1(17^+^)Lox contains an intact UL23 ORF. Between UL22 and UL23, the BAC genes and a eukaryotic Cre recombinase expression cassette are flanked by loxP sites. Thus, after reconstitution of the virus in cell culture these non-viral sequences are excised, leaving a single loxP site as the only foreign sequence; furthermore HSV-1(17^+^)Lox lacks, as other HSV1-BACs propagated in *E. coli* [1], the oriL that is present in the wild-type strain HSV-1(17^+^).

A firefly luciferase gene under control of a human CMV promoter was inserted between the UL55 and UL56 ORFs. To this end, the expression cassette together with a FRT flanked kanamycin resistance was amplified from pC3lucM157-Kan [7] using primers nm0003-1 and nm0002-1 (Table S1) and inserted into HSV1(17^+^)Lox via Red-recombination. Expression of Flp recombinase from pCP20 removed the kanamycin resistance. The resulting virus was named HSV1(17^+^)Lox-Luc.

**Deleting the HSV-1 neurovirulence factor γ_1_34.5 and inserting a LIF expression cassette.** To reduce neurovirulence of the HSV-1 vectors, we removed both copies of the γ_1_34.5 gene from the terminal repeats flanking the U_L_ region of the HSV-1 genome [8]. Starting with HSV-1(17^+^)Lox-Luc, first the 3' copy of γ_1_34.5 (nucleotides 125112-125858 in wt HSV-1(17^+^); GenBank NC_001806) was replaced by a kanamycin resistance cassette flanked by mFRT sites which was amplified from pOri6K-F5 [9] with the primers ICP34.5.rev and ICP34.5.for (Table S1). Subsequently, the kanamycin resistance was removed by Flp recombinase, leaving one mFRT site in the HSV-1 genome. Likewise, the 5' copy of γ_1_34.5 (nucleotides 603-1259 of wt HSV-1(17^+^) was replaced with a Zeocin resistance cassette (ZeoR) which was amplified from pZeo4 (kindly provided by Martin Messerle, Hannover, Germany) with the primers ICP34.5zeo.for and ICP34.5zeo.rev (Table S1). The resulting virus was named HSV-1(17^+^)Lox-Luc-Δγ_1_34.5-Zeo or for short, HSV-Zeo.

To construct an HSV-1 vector expressing LIF, the ZeoR gene in the 5' copy of γ_1_34.5 in HSV-Zeo was replaced with LIF (under the EF1α promoter) using the *en passant* mutagenesis protocol [10,11]. A kanamycin resistance cassette (KanR) together with 50 bp of a homologous sequence of EF1α-LIF and an I-SceI-site were amplified with primers LIF-KanL2 and LIF-KanR (Table S1) from the plasmid pGP704-Kan [12]. The PCR product and pcDNA3.1-EF1αLIF were cut with BamHI and ligated, yielding the plasmid pcDNA3.1-EF1αLIF-Kan. Thereafter, the EF1α, LIF, I-SceI-site, KanR and the homologous sequences were amplified with primers LIF-ICP34.5-L and LIF-ICP34.5-R (Table S1), containing approximately 80 bp overhanging sequences complementary to sequences upstream and downstream of the insertion site (nucleotides 603-1259 of the γ_1_34.5 gene in HSV-1(17^+^)). The PCR product was used to transform electrocompetent *E. coli* strain GS1783 [11] containing HSV-Zeo. To remove the kanamycin resistance cassette, I-SceI was induced by L-arabinose and the Red enzyme expression was induced by heat shock. The bacteria were selected for with L-arabinose and chloramphenicol and kanamycin-sensitive clones were selected for restriction analysis. The resulting virus was named HSV-1(17^+^)Lox-Luc-Δγ_1_34.5-LIF or for short, HSV-LIF.

**Supplementary references:**

1. Nagel CH, Döhner K, Fathollahy M, Strive T, Borst EM, et al. (2008) Nuclear egress and envelopment of herpes simplex virus capsids analyzed with dual-color fluorescence HSV1(17+). J Virol 82: 3109-3124.

2. Lee EC, Yu D, Martinez de Velasco J, Tessarollo L, Swing DA, et al. (2001) A highly efficient Escherichia coli-based chromosome engineering system adapted for recombinogenic targeting and subcloning of BAC DNA. Genomics 73: 56-65.

3. Gu H, Marth JD, Orban PC, Mossmann H, Rajewsky K (1994) Deletion of a DNA polymerase beta gene segment in T cells using cell type-specific gene targeting. Science 265: 103-106.

4. Smith GA, Enquist LW (2000) A self-recombining bacterial artificial chromosome and its application for analysis of herpesvirus pathogenesis. Proc Natl Acad Sci U S A 97: 4873-4878.

5. Cherepanov PP, Wackernagel W (1995) Gene disruption in Escherichia coli: TcR and KmR cassettes with the option of Flp-catalyzed excision of the antibiotic-resistance determinant. Gene 158: 9-14.

6. Datsenko KA, Wanner BL (2000) One-step inactivation of chromosomal genes in Escherichia coli K-12 using PCR products. Proc Natl Acad Sci U S A 97: 6640-6645.

7. Klenovsek K, Weisel F, Schneider A, Appelt U, Jonjic S, et al. (2007) Protection from CMV infection in immunodeficient hosts by adoptive transfer of memory B cells. Blood 110: 3472-3479.

8. Chou J, Kern ER, Whitley RJ, Roizman B (1990) Mapping of herpes simplex virus-1 neurovirulence to gamma 134.5, a gene nonessential for growth in culture. Science 250: 1262-1266.

9. Borst EM, Messerle M (2005) Analysis of human cytomegalovirus oriLyt sequence requirements in the context of the viral genome. J Virol 79: 3615-3626.

10. Tischer BK, von Einem J, Kaufer B, Osterrieder N (2006) Two-step red-mediated recombination for versatile high-efficiency markerless DNA manipulation in Escherichia coli. Biotechniques 40: 191-197.

11. Tischer BK, Smith GA, Osterrieder N (2010) En passant mutagenesis: a two step markerless red recombination system. Methods Mol Biol 634: 421-430.

12. Loewendorf A, Krüger C, Borst EM, Wagner M, Just U, et al. (2004) Identification of a mouse cytomegalovirus gene selectively targeting CD86 expression on antigen-presenting cells. J Virol 78: 13062-13071.
